# Recent Progress Concerning the *N*-Arylation of Indoles

**DOI:** 10.3390/molecules26165079

**Published:** 2021-08-22

**Authors:** Petr Oeser, Jakub Koudelka, Artem Petrenko, Tomáš Tobrman

**Affiliations:** Department of Organic Chemistry, University of Chemistry and Technology, 16628 Prague, Czech Republic; petr.oeser@vscht.cz (P.O.); jakub.koudelka@vscht.cz (J.K.); petrenka@vscht.cz (A.P.)

**Keywords:** *N*-arylation, indole, Buchwald–Hartwig amination, Ullmann condensation, Chan–Lam coupling

## Abstract

This review summarizes the current state-of-the-art procedures in terms of the preparation of *N*-arylindoles. After a short introduction, the transition-metal-free procedures available for the *N*-arylation of indoles are briefly discussed. Then, the nickel-catalyzed and palladium-catalyzed *N*-arylation of indoles are both discussed. In the next section, copper-catalyzed procedures for the *N*-arylation of indoles are described. The final section focuses on recent findings in the field of biologically active *N*-arylindoles.

## 1. Introduction

**Figure 1:**
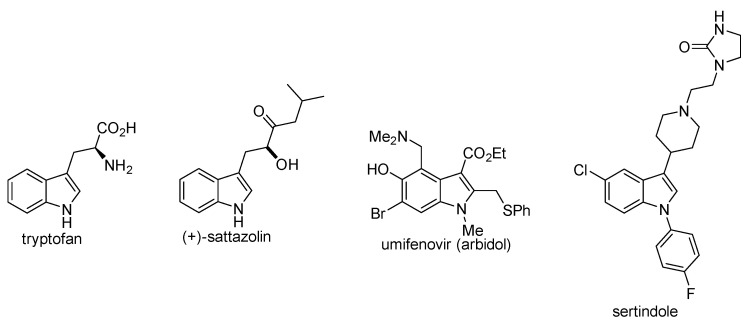
Selected examples of important indole derivatives.

Heterocyclic compounds are cyclic compounds that each contain either a heteroatom or a number of heteroatoms. Among the most important groups of heterocyclic compounds are the aromatic heterocyclic compounds. Well-known examples of such compounds include pyridine, pyrrole, thiophene, and furan. Indoles occupy a unique position within the field of heterocyclic compounds. An indole is a condensed compound that is composed of a benzene and pyrrole core. The importance of indoles and their derivatives is illustrated by the examples shown in Figure 1. Tryptophan is an aromatic essential amino acid, and sattazolin [1] and umifenovir [2] are indole derivatives with antiviral effects. *N*-arylated indoles also belong to the group of important indole derivatives. An example of such a compound is sertindole, which is used as an antipsychotic drug [3]. Due to the significant biological activity of the indole derivatives, much attention has been paid to the modification of the indole unit [4,5,6,7,8]. 

**Scheme 1:**
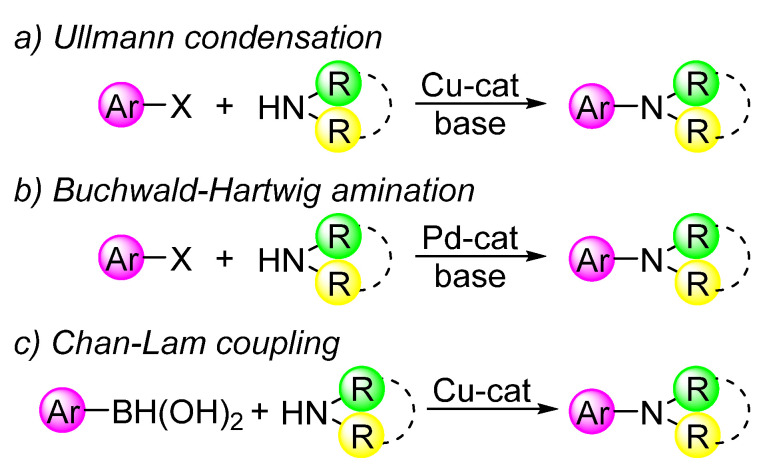
Transition-metal-catalyzed approaches to *N*-arylamines.

The significant biological activity exhibited by sertindole has inspired the search for new procedures for the preparation of *N*-arylindoles. Every indole contains a pyrrole core, so it can be expected that the *N*-arylation of indoles will proceed in a similar manner to the *N*-arylation of pyrroles. Among the group of aromatic heterocyclic compounds with one heteroatom, pyrrole is unique in terms of its reactivity. Unlike pyrrole, modifications of heteroatoms in furan and thiophene to form stable derivatives of the starting compound are not feasible. In some cases, phospholes can be *P*-arylated to form pentasubstituted phospholes [9], although the reactivity of the phosphole is affected by its low aromaticity [10,11,12,13]. By contrast, pyrroles can be very easily arylated at position 1 [14,15] or 2 [16,17]. In addition, the dearomatizations of pyrroles are important [18,19,20,21,22]. Similar reactivity can be expected even in the case of indole arylations. The typical approach for the *N*-arylation of amines, including nitrogen-containing heteroaromatic compounds, involves the use of transition-metal-catalyzed reactions (Scheme 1). In these cases, Ullman condensation [23,24,25], Buchwald–Hartwig amination [26,27,28], and Chan–Lam coupling [29,30,31] are all frequently used. A common feature of these procedures is the reaction of amines with organohalides or boronic acids in the presence of a transition metal catalyst and a base. A relatively large number of *N*-arylation reactions of indoles have been reported over the last decade. However, to the best of our knowledge, only a few review articles have addressed the preparation of *N*-arylindoles [32,33,34]. Thus, we decided to summarize the procedures that have been used for the preparation of *N*-arylindoles over the past decade. The scope of the present review is limited to works that focused on the *N*-arylation of indoles or otherwise significantly contributed to this field.

## 2. Transition-Metal-Free *N*-Arylation of Indoles

**Scheme 2:**
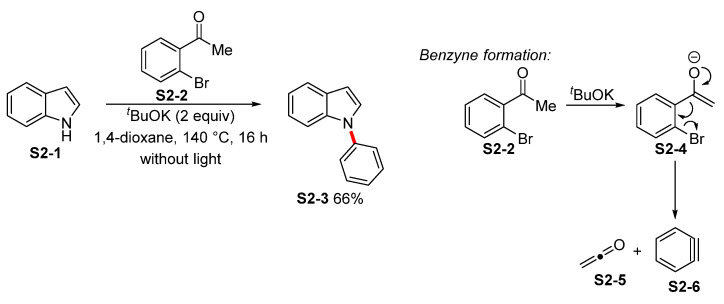
*N*-arylation of an indole using a benzyne intermediate.

The preparation of *N*-arylindoles without transition metal catalysts can be easily carried out using benzynes. This approach was first reported in 2018 (Scheme 2) [35]. In this study, the starting indole **S2-1** was treated with 2-bromoacetophenone (**S2-2**) in the presence of *^t^*BuOK to produce *N*-phenylindole **S2-3** in a 66% yield. The proposed mechanism accounts for the formation of a benzyne intermediate (**S2-6**) and ketene (**S2-5**) from the enolate **S2-4**. The scope of the reaction is limited to the indole **S2-3**. However, Chen and Wu published another benzyne-involving route to functionalized indoles, which in contrast, furnishes mainly C3-arylated indole derivatives, along with *N*-arylated indoles as side-products [36].

**Scheme 3:**
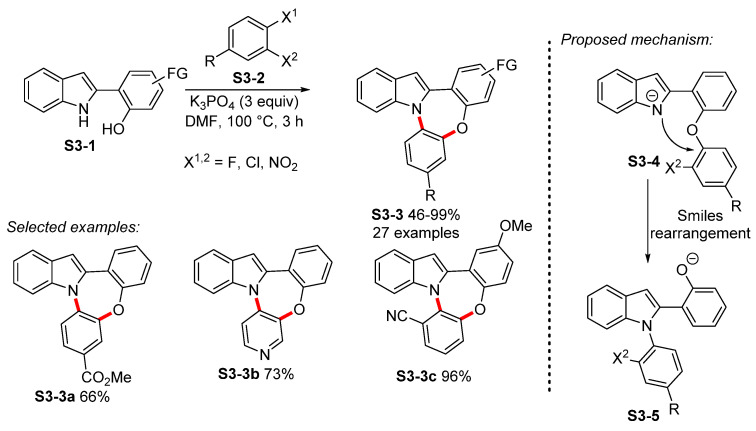
Preparation of dibenzo[b,f][1,4]oxazepines **S3-3** by means of Smiles rearrangement.

The second and seemingly most frequently used approach is based on nucleophilic aromatic substitution. The intramolecular *N*-arylation of indoles **S3-1** was used for the preparation of dibenzo[b,f][1,4]oxazepines **S3-3** (Scheme 3) [37]. Based on the general reaction, it appears that the methodology relies on Smiles rearrangement. This assumption has been experimentally confirmed, and the proposed mechanism assumes the formation of diaryl ether **S3-4**, which is converted into the *N*-arylated indole **S3-3** by means of the Smiles rearrangement. Selected examples (**S3-3a–S3-3c**) show that the described procedure works in relation to the preparation of pyridine derivatives of **S3-3b** and in the case of the regioisomer **S3-3c** of the starting materials **S3-2**.

**Scheme 4:**
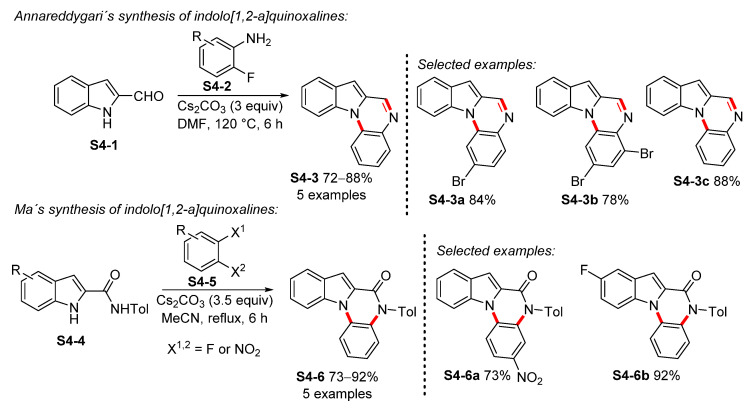
Transition-metal-free syntheses of indolo[1,2-a]quinoxalines.

A similar concept for the *N*-arylation of indoles, which is based on intramolecular nucleophilic aromatic substitution, has been described by both Annareddygari [38] and Chen Ma [39] (Scheme 4). The first procedure for the preparation of indolo[1,2-a]quinoxalines **S4-3** makes use of the condensation reaction of 2-formylindole **S4-1** with substituted anilines **S4-2** in *N*,*N*-dimethylformamide (DMF) at 120 °C. Similarly, the condensation–intramolecular-based transition-metal-free arylation approach was used by Ngernmeesri for the synthesis of indolo[1,2-a]quinolines [40]. Ma’s procedure starts with amides **S4-4**, and the *N*-arylation of the indole is achieved via double intramolecular nucleophilic aromatic substitution. In both cases, the scope of the studied reaction was limited to the preparation of five indole derivatives, **S4-6**. Intramolecular nucleophilic aromatic substitution has also been used for the preparation of 9*H*-pyrrolo[1,2-a]indol-9-ones and 10*H*-indolo[1,2-a]indol-10-ones by means of the base-mediated *N*-arylation of pyrroles and indoles [41].

**Scheme 5:**
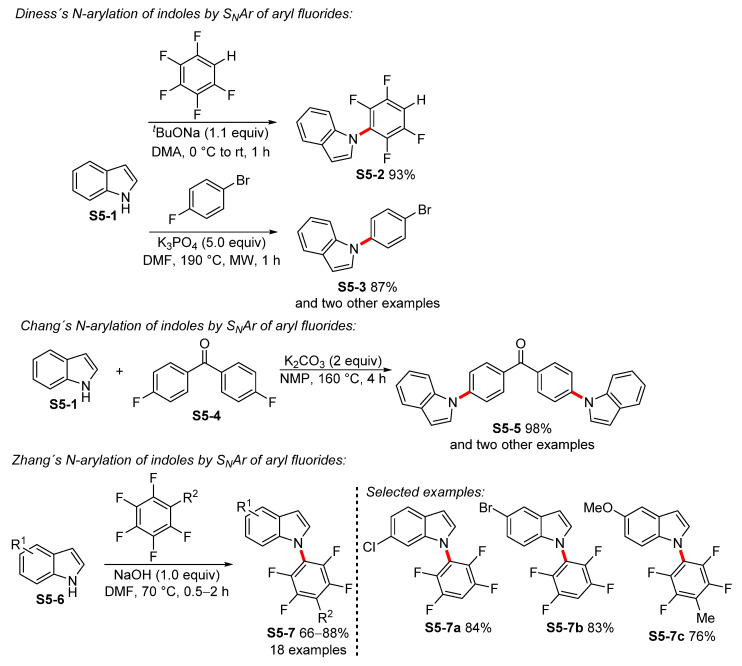
Intermolecular nucleophilic aromatic substitution for the synthesis of *N*-arylindoles.

Intermolecular nucleophilic aromatic substitution can also be used to prepare *N*-arylindoles (Scheme 5). This concept for the preparation of *N*-arylindoles is usually based on the reaction of aryl fluorides with indoles under basic conditions. Diness et al. developed two procedures for the preparation of *N*-arylindoles. In the first procedure, the reaction of pentafluorobenzene with an indole in the presence of *^t^*BuONa afforded *N*-arylindole **S5-2**, which was then used for the preparation of pentasubstituted benzene (Scheme 5, Diness’s *N*-arylation of indoles) [42]. The analogous reaction with a halogenated aromate containing only one fluorine atom proceeded under harsh reaction conditions, as illustrated by the selected synthesis of indole **S5-3**. However, the overall reaction scope was limited to three examples of *N*-arylated indoles [43]. Similar reaction conditions were used for the preparation of bis *N*-arylindoles with structures represented by the indole **S5-5** (Scheme 5, Chang’s *N*-arylation of indoles). The prepared *N*-arylindoles were polymerized, and their optical and electrochemical properties and thermal stability were determined [44]. The results summarized in Scheme 5 indicate that the use of nucleophilic aromatic substitution for the preparation of *N*-arylindoles proceeds best in the case of pentafluorobenzenes. This conclusion was confirmed by the work of Zhang [45], who prepared a series of tetrafluorophenylbenzenes through the reaction of indoles with pentafluorobenzenes in the presence of sodium hydroxide under mild reaction conditions (Scheme 5, Zhang’s *N*-arylation of indoles). Similar reaction conditions (KOH, DMSO, 100 °C, 24 h) were used by Hua to prepare a series of *N*-arylated indoles (23 compounds, 24–87% isolated yields) [46].

In addition to the above-mentioned procedures for the transition-metal-free arylation of indoles by means of intermolecular nucleophilic aromatic substitution, other procedures have also been used to achieve limited *N*-arylation of indoles as parts of general procedures for the preparation of *N*-arylated amines. Such works include the preparation of benzimidazole-pyrrolo[1,2-a]quinoxaline [47], arene-metal π-complexation mediated C–H arylation [48], base-promoted nucleophilic fluoroarene substitution of C–F bonds [49], and the preparation *N*-arylation of amines via triarylsulfonium triflates [50].

**Scheme 6:**
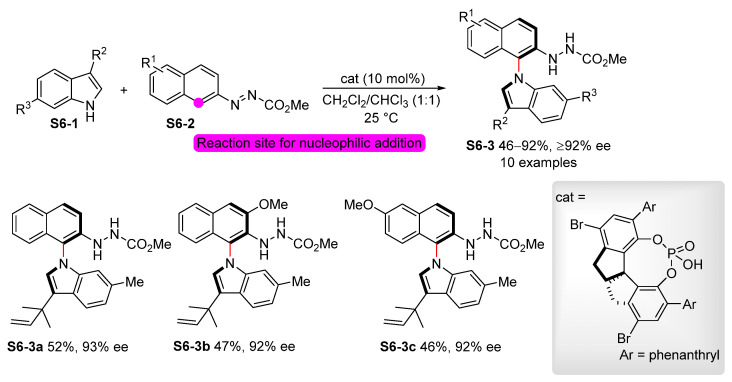
Atroposelective *N*-arylation of indoles by arene C–H amination.

The organocatalytic preparation of *N*-aryl carbazoles and indoles was reported in 2020 (Scheme 6) [51]. The reaction between the azonaphthalene **S6-2** and *N*-nucleophiles was catalyzed by chiral phosphoric acid derivatives. Although the majority of reported *N*-arylations have been performed on carbazole (26 examples), the reaction has also been used for the preparation of ten *N*-arylindoles. It was proposed that the formation of the reaction products **S6-3** involves the asymmetric nucleophilic addition of indoles **S6-1** to the reaction center, and that the final product of the reaction **S6-3** is obtained via rearomatization. Unfortunately, the scope of the reaction was limited to azonaphthalene **S6-2**, and the scope of the indoles was also limited.

**Scheme 7:**
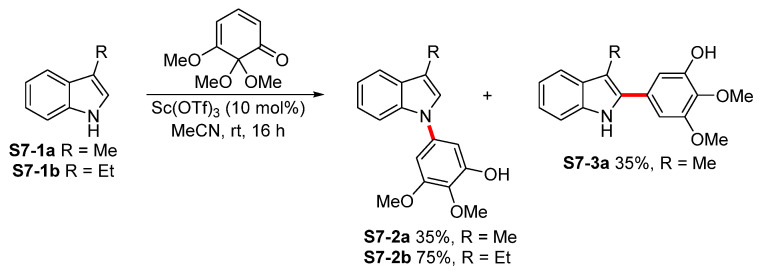
Synthesis of *N*-arylindoles via an aza-Michael addition and aromatization sequence.

A different approach to the *N*-arylation of indoles, which involves aza-Michael addition and aromatization, was reported in 2018 (Scheme 7) [52]. The developed methodology is based on the reaction of azoles with cyclohexa-2,4-dienones in the presence of a catalytic amount of scandium triflate in acetonitrile. The procedure can be used for the regioselective preparation of *N*-aryl pyrazoles, 1,2,4-triazoles, benzimidazoles, and 1,2,3-benzotriazoles in high yields. In the case of substituted indoles, the low selectivity of the arylation reaction was observed. Thus, 3-methylindole **S7-1a** reacted with the starting dienone to form a mixture of C2- and *N*-arylated indoles **S7-2a** and **S7-3a**. By contrast, 3-ethylindole **S7-1b** exclusively gave *N*-arylindole **S7-2b** in a 75% yield. The observed reactivity was not explained.

**Scheme 8:**
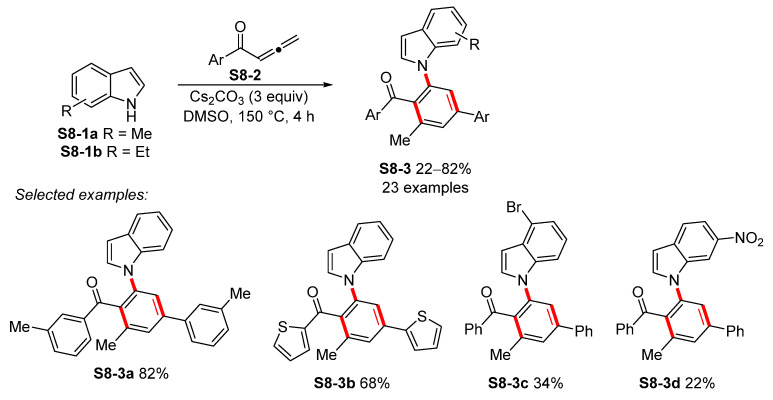
Cascade benzannulation for the synthesis *N*-arylated indoles.

The transition-metal-free synthesis of *N*-arylindoles **S8-3** from 1,2-allenic ketones **S8-2** and variously substituted indoles **S8-1** via a Cs_2_CO_3_-promoted tandem benzannulation reaction has recently been demonstrated by Li et al. (Scheme 8) [53]. The substrate scope with respect to both indoles **S8-1a** and **S8-1b** and allenic ketones **S8-2** is quite broad. Interestingly, the C2-substituted indoles were either unreactive or reacted poorly under the reaction conditions, likely due to steric hindrance. A mechanism that involves a series of Michael additions followed by cyclization and benzannulation may be operative, as proposed by the authors.

## 3. Transition-Metal-Catalyzed *N*-Arylation of Indoles

### 3.1. Nickel-Catalyzed N-Arylation of Indoles

**Scheme 9:**
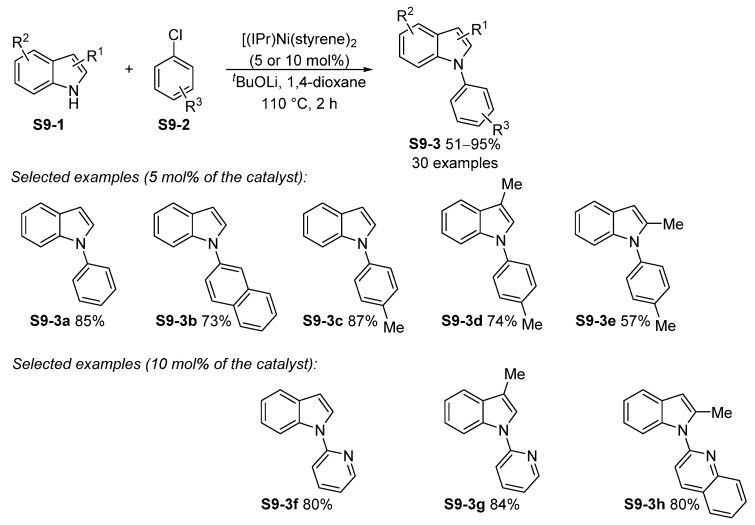
Scope of the *N*-arylation of indoles with aryl chlorides catalyzed by [(IPr)Ni(styrene)_2_].

Nickel(0) complexes are highly reactive toward oxidative addition, and therefore, nickel catalysts are used for the cross-coupling reactions of aryl chlorides. Rull [54] was the first to report the Ni(0)-catalyzed arylation of indole **S9-1** using aryl chlorides **S9-2** (Scheme 9). The *N*-arylations were carried out using the [(IPr)Ni(styrene)_2_] complex [IPr=1,3-Bis(2,6-diisopropylphenyl)imidazol-2-ylidene] in the presence of *^t^*BuOLi in 1,4-dioxane at 100 °C. As illustrated by selected examples **S9-3a**–**S9-3h**, the yields of the *N*-arylindoles were high. No reaction was observed with aryl chlorides bearing trifluoromethyl and benzoyl groups or *ortho*-substituted chlorobenzenes. Yet, the preparation of indoles with heteroaryl substituents required 10 mol% of the catalyst loading to achieve high yields of the corresponding indoles **S9-3g** and **S9-3h**. The need to use 10 mol% of the catalyst was explained by the competitive coordination of heteroaryl halide during the *N*-arylation reaction.

**Scheme 10:**
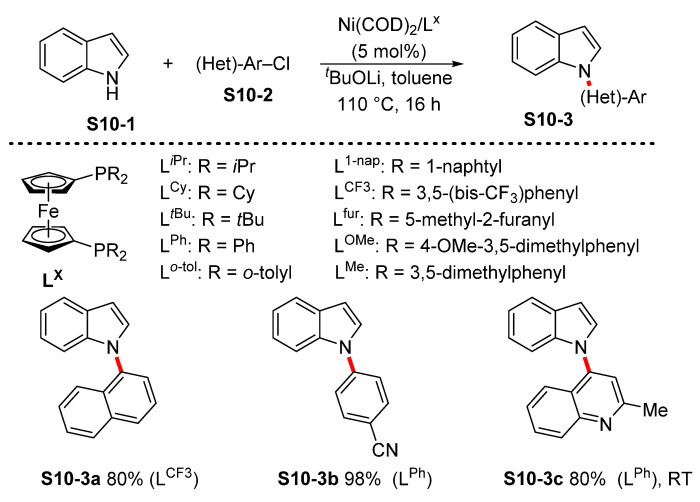
Comparative catalytic screening during the nickel-catalyzed *N*-arylation of indoles.

Different catalytical systems for the *N*-arylation of indoles were studied by Stradiotto [55], who investigated the reactivity of DPPF-based ligands (Scheme 10) during the Ni(0)-catalyzed *N*-arylation of indoles (Scheme 10). Heteroaryl chlorides were coupled with alkylamines and indole in the presence of ten structurally diverse 1,1′-bis(bis(alkyl/aryl)phosphino)ferrocene ligands (L^X^) and Ni(COD)_2_. The electron-poor ligand L^CF3^ proved to be extremely potent. It was the only ligand that gave the *N*-arylated indole **S10-3a** bearing a naphthyl substituent in a 80% yield. By contrast, the *N*-arylation of an indole with 4-chlorobenzonitrile proceeded efficiently with several L^X^ variants, including L*^i^*^Pr^, L^Cy^, L^Ph^, L^CF3^, L^OMe^, and L^Me^. The amination of 4-chloroquinaldine with the ligand L^Ph^ represents the first room-temperature *N*-arylation of an indole. In addition, ligands with *ortho*-substituted phenyl (L^o-tol^ and L^1-nap^), a sterically demanding dialkylphosphino group (L*^t^*^Bu^), and difuranylphosphino variants (L^fur^) proved ineffective under the studied conditions. The comparable catalytic performance of L^Ph^, L^CF3^, and L^OMe^ in the studied nickel-catalyzed *N*-arylations suggests that the electronic perturbations arising from arylphosphine substitution do not significantly affect the behavior of nickel in this branch of chemistry.

**Scheme 11:**
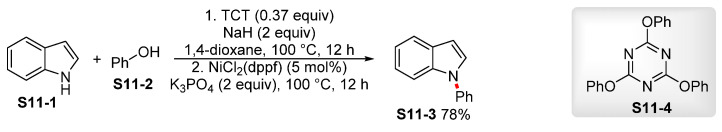
Ni-catalyzed *N*-arylation of phenols via C–O bond activation by means of a TCT reagent.

A different procedure for *N*-arylation is based on the reaction of amines or heterocyclic compounds with phenols (Scheme 11) [56]. Phenols are reluctant to react during cross-coupling reactions, so the starting phenol is converted into a cyanuric acid ester **S11-4** via the reaction with cyanuric chloride (TCT) in 1,4-dioxane in the presence of a base. The conditions for the formation of the cyanuric acid ester **S11-4** were obtained through extensive optimization. For example, the *N*-arylation of indole **S11-1** with activated phenol **S11-4** was performed using the NiCl_2_(dppf) catalytic system to give the *N*-arylated indole **S11-3** in a 78% yield. Morpholine, carbazole, indoline, and pyrazole are examples of the other tested amines.

**Scheme 12:**
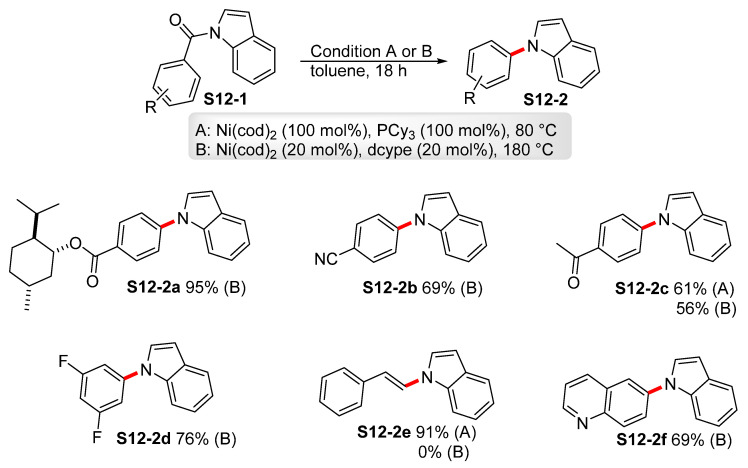
Ni-catalyzed decarbonylation of *N*-acylated heteroarenes.

The nickel-catalyzed decarbonylation of substituted *N*-acyl indoles **S12-1** represents another approach for the preparation of *N*-arylated indoles (Scheme 12) [57]. Two conditions were optimized for the Ni(0)-catalyzed decarbonylation of the starting indoles. First, stoichiometric amounts of Ni(cod)_2_ and PCy_3_ preferred quantitative decarbonylation at 80 °C (Condition A). Second, catalytic decarbonylation was achieved through the use of the 1,2-ethanediylbis[dicyclohexyl]phosphine (dcype) ligand at 180 °C (Condition B). Condition B proved to be suitable for *N*-acylindoles bearing an electron withdrawing group, whereas Condition A was suitable for *N*-acylindoles bearing an acyl group with electron-neutral and electron-rich substituents. Selected examples **S12-2a–S12-2f** indicate that this method tolerates esters, nitriles, and the ketone functional group. In addition to benzoic acid derivatives, this method has proven suitable for the formation of *N*-alkenylindoles **S12-2e** and *N*-heteroarylindoles **S12-2f**.

**Figure 2:**
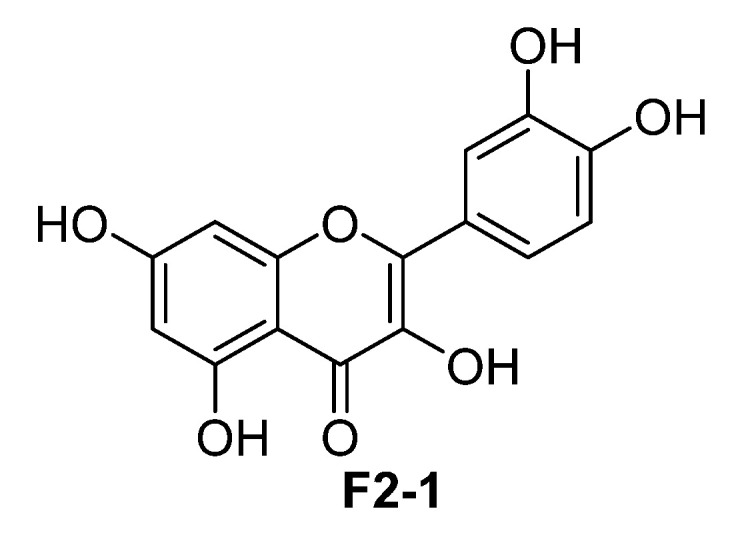
The structure of quercetin.

Recently, the use of NiO nanoparticles for the *N*-arylation of indoles by means of the reaction of boronic acids with indoles in the presence of NiO nanoparticles as a catalyst has been reported [58]. Pure NiO nanoparticles were prepared using the quercetin-mediated (**F2-1**, Figure 2) hydrothermal method.

**Figure 3:**
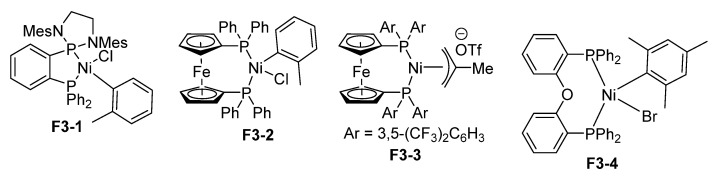
Examples of nickel catalysts used for the *N*-arylation of amines.

In addition to the above-mentioned nickel-based catalytic systems for the *N*-arylation of indoles, the use of nickel catalysts **F3-1** [59], **F3-2** [60], **F3-3** [61], **F3-4** [62], and Ni(COD)_2_ with a polymeric triazine-phosphite ligand [63] for the reaction of amines or heterocycles with electrophiles has also been reported (Figure 3). A unique procedure for the preparation of *N*-aryl amines and indoles makes use of the nickel-catalyzed decarbonylative amination of carboxylic acid esters [64]. However, such procedures have mainly been developed for the *N*-arylation of amines, whereas the *N*-arylation of indoles has been achieved for only a limited number of indole derivatives.

### 3.2. Palladium-Catalyzed N-Arylation of Indoles

**Scheme 13:**
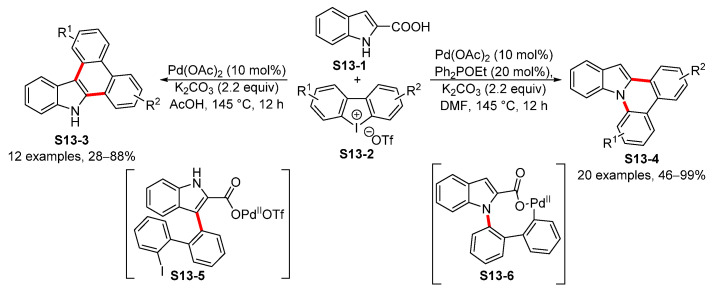
Palladium-catalyzed site-selective N1/C2 and C3/C2 arylation of indoles.

Similarly to the palladium-catalyzed arylation of pyrroles, the formation of 1, 2, and 3-arylated indoles can also be observed in the palladium-catalyzed arylation of indoles (Scheme 13) [65,66,67]. The regioselectivity of palladium-catalyzed indole arylation can be influenced by the detailed optimization of the reaction conditions. This was demonstrated in 2019 by Zhang et al., who reported the palladium-catalyzed cascade decarboxylative arylation of indoles [68]. The authors optimized the course of the reaction of indolyl carboxylic acids **S13-1** with diaryliodonium salts **S13-2** and determined that N1/C2 or C2/C3 arylations of indoles can be performed with high regioselectivity. The reaction allows the introduction of variously substituted biphenyls. The proposed key intermediates for both C2/C3 and N1/C2 arylation, **S13-5** and **S13-6**, show that the *N*-arylation of indoles is the first step in N1/C2 arylation. However, the C3 arylation of the starting material occurs first in the case of C2/C3 arylation of the starting indolyl carboxylic acid **S13-1**.

**Scheme 14:**
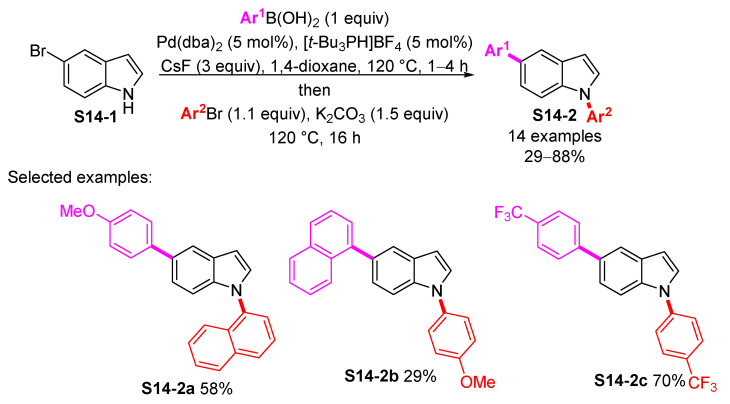
Sequential Suzuki arylation and Buchwald–Hartwig amination for the synthesis of *N*-arylated indoles.

A different approach for the preparation of diarylated indoles was reported in 2020 (Scheme 14) [69]. Muller et al. used 5-bromoindole to prepare diarylated indoles via sequential Suzuki reaction and Buchwald–Hartwig amination. The Suzuki reaction was performed first to avoid any side reactions. The subsequent amination reaction uses the same catalytic system as the Suzuki reaction, although potassium carbonate is used instead of CsF. Selected examples **S14-2a** and **S14-2b** illustrate how the Suzuki reaction of bromoindole **S14-1** with 1-naphthylboronic acid gives worse yields of the indole **S14-2b** than the Buchwald–Hartwig amination with naphthyl bromide. This approach is also suitable for the preparation of diarylated carbazoles and 10*H*-phenothiazines.

**Scheme 15:**
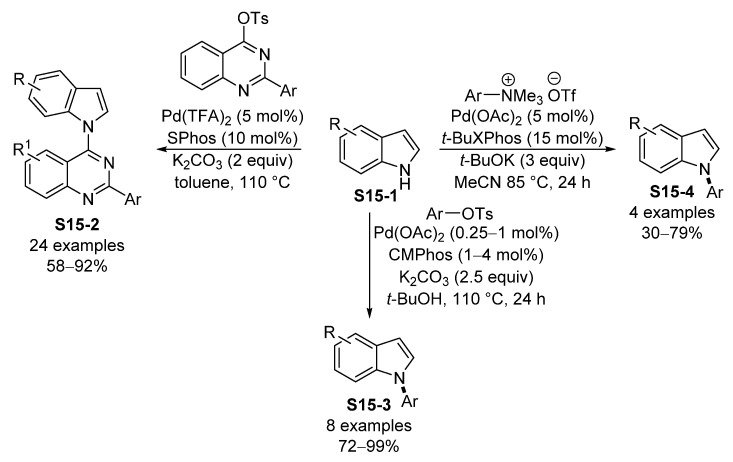
Palladium-catalyzed *N*-arylation of indoles via the reactions with substrates with activated C–O and C–N bonds.

An alternative approach to *N*-arylated indoles utilizes reactions the substituted indole **S15-1** with substrates with activated C–O and C–N bonds. Selected examples are presented in Scheme 15. In some cases, the palladium-catalyzed reaction of quaternary ammonium salts with indoles in the presence of potassium *tert*-butoxide can be used to prepare *N*-arylated indoles, although the reported scope of the reaction is limited to the preparation of four simple *N*-arylindoles, **S15-4**. However, the synthesis of *N*-alkylated indoles by means of this approach is convenient [70]. Additionally, tosylates are sufficiently reactive for the preparation of *N*-arylindoles. In this way, a large group of quinazolines **S15-2** were prepared in high isolated yields, although similar nonaflates do not react under the developed reaction conditions [71]. A similar approach for the synthesis of *N*-arylindoles **S15-3** was reported in 2018 by Kwong, albeit with a significantly narrower scope [72].

**Scheme 16:**
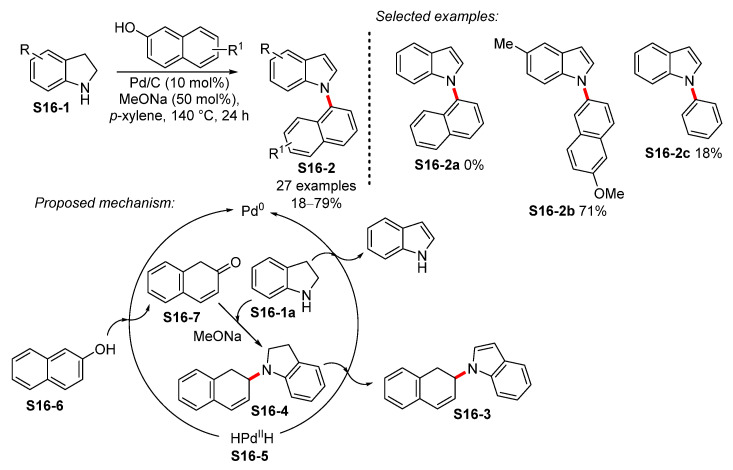
Hydrogen-transfer-mediated *N*-arylation of indolines en route to *N*-arylated indoles.

A hydrogen-transfer-mediated cross-coupling reaction can also be used for the preparation of *N*-arylindoles (Scheme 16) [73]. The starting material, as represented by the structure of substituted indolines **S16-1**, reacts with 2-naphthols and their derivatives in the presence of Pd/C and sodium methoxide. Optimized conditions were applied to prepare 27 indole derivatives **S16-2** with a naphthyl group at position 1. The reaction can tolerate, for example, an ester and a hydroxy functional group. However, 1-naphthols do not react under the optimized reaction conditions, as illustrated in Scheme 16. The proposed mechanism assumes the formation of active catalyst **S16-5** by means of the reaction of Pd(0) with indoline. The resultant palladium catalyst **S16-5** subsequently hydrogenates 2-naphthol **S16-6** to form the intermediate **S16-7**. The final reaction product is formed by the condensation of intermediate **S16-7** with indoline **S16-1a**, followed by palladium-mediated dehydrogenation.

**Scheme 17:**
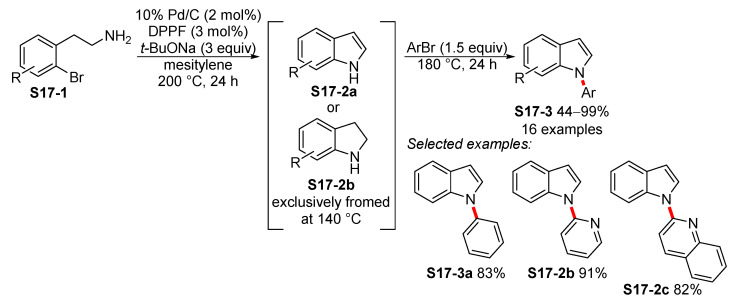
Palladium-catalyzed one-pot synthesis of *N*-arylated indoles.

The two-step one-pot procedure for the preparation of *N*-arylindoles relies on the palladium-catalyzed cyclization of 2-bromophenethylamines **S17-1**, which is followed by the palladium-catalyzed *N*-arylation of the resulting indoles **S17-2a** (Scheme 17) [74]. The optimization of the reaction conditions revealed that the cyclization of amines **S17-1** into indoles **S17-3** must be carried out at 200 °C. Running the same cyclisation reaction at 140 °C exclusively produces indoline derivatives **S17-2b** and intermediates. In the next step, the arylation of the resulting indole **S17-2a** is performed using the same catalytic system at 180 °C.

**Scheme 18:**
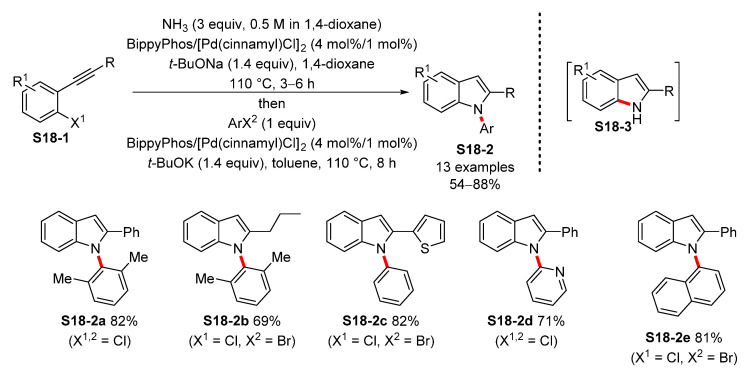
Double *N*-arylation for the preparation of *N*-arylated indoles.

A similar approach for the preparation of *N*-arylated indoles was reported by Stradiotto (Scheme 18) [75]. In this case, the starting alkynes **S18-1** reacts with ammonia to form indole-based intermediates **S18-3**. In the next step, the *N*-arylation of the resulting indole is performed using the same catalytic system in the presence of potassium *tert*-butoxide. This procedure led to the preparation of 13 indole derivatives. From the selected examples **S18-2a–S18-2e**, it is evident that the *N*-arylation of indoles also tolerates *ortho*-disubstituted aryl halides. The reaction conditions for the *N*-arylation of indoles developed in relation to the two-step one-pot procedure can also be used for the *N*-arylation of independently prepared indoles.

**Scheme 19:**
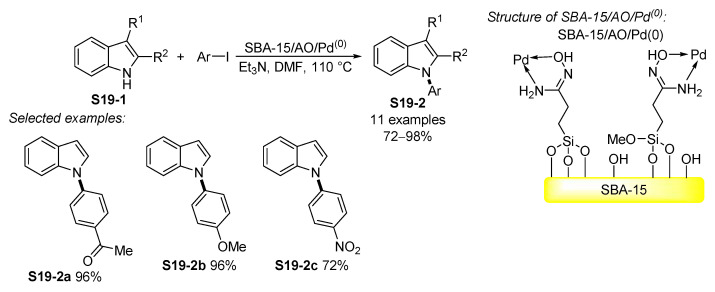
Amidoxime-functionalized mesoporous SBA-15 as a heterogeneous and recyclable nanocatalyst for indole *N*-arylation.

Recently, a series of papers have been published on the *N*-arylation of indoles using palladium catalysts immobilized on mesoporous silica SBA-15 [76,77], carbon nanotubes [78], or magnetic Fe_3_O_4_ nanoparticles [79]. Scheme 19 illustrates the immobilization of the palladium catalyst on the surface of SBA-15 through the coordination of palladium to the amidoxime group [76]. The major advantages offered by immobilized catalysts are their recyclability and reusability. This was demonstrated by reusing the immobilized catalyst in five catalytic cycles [76].

**Scheme 20:**
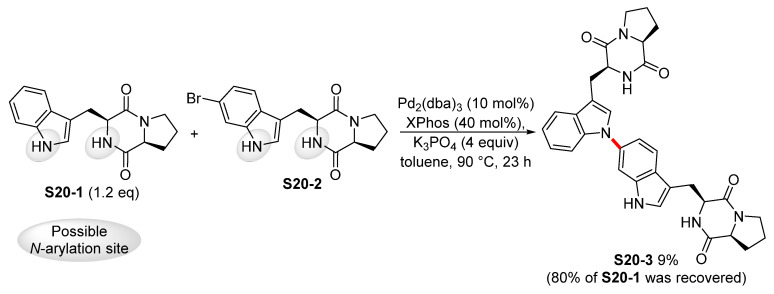
Use of *N*-arylation of indoles for the preparation of (–) Aspergilazine A.

Palladium-catalyzed *N*-arylation of indoles has been used for the total synthesis of (–)-aspergilazine A (Scheme 20) [80]. In this report, the preparation of (–)-aspergilazine A (**S20-3**) was achieved by means of a cross-coupling reaction between non-brominated **S20-1** and brominated indole **S20-2** to form (–)-aspergilazine A (**S20-3**) at a 9% yield. When evaluating this approach to (–)-aspergilazine A, it should be noted that the starting substances contained four N–H bonds that could be arylated. In addition, 80% of the unreacted starting material **S20-1** was isolated together with the reaction product **S20-3**.

**Figure 4:**
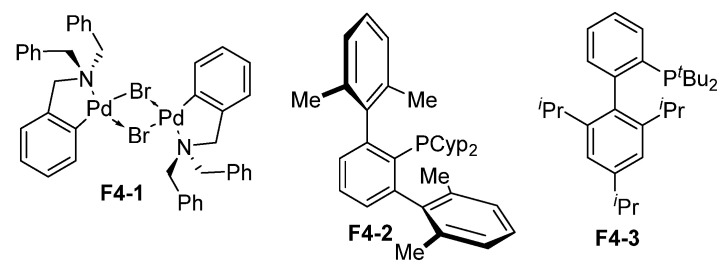
Selected examples of general palladium-catalyzed *N*-arylations used for the preparation of *N*-arylindoles.

Other examples of Buchwald–Hartwig indole *N*-arylation include the use of the ligands **F4-1** [81], **F4-2** [82], and *t*BuXphos (**F4-3**) [83] (Figure 4). In addition, the palladium-catalyzed amination of triflates [84,85]; cross-coupling reactions of silylated indoles with aryl halides [86,87]; and reactions that are catalyzed by Pd/ZnO nanoparticles [88], palladium immobilized on graphene [89], or silica-starch substrates [90] were used to prepare *N*-arylindoles. However, these procedures have mainly been developed for the *N*-arylation of amines, whereas the *N*-arylation of indoles was given for only a limited number of indole derivatives.

### 3.3. Copper-Catalyzed N-Arylation of Indoles

**Scheme 21:**
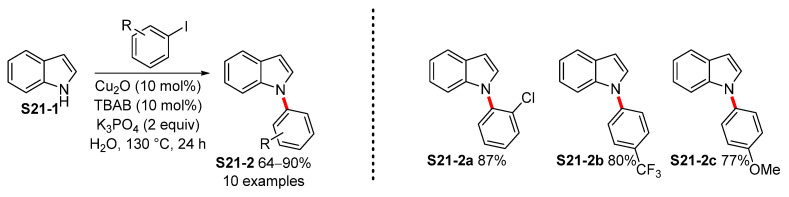
Ligand-free copper-catalyzed *N*-arylation of indole.

Ligand-free cross-coupling reactions can be considered economical and advantageous due to the cost reductions associated with the designed chemical transformations. In addition, indole *N*-arylations have been the subjects of ligandless experimental setups. This approach for the preparation of *N*-aryl azoles, including *N*-arylindoles, was reported by Teo in 2011 (Scheme 21) [91]. The reaction of unsubstituted indoles with aryl iodides in the presence of catalytic amounts of copper oxide and tetrabutylammonium bromide (TBAB) as phase-transfer catalysts in water gave *N*-arylindoles **S21-2** in high isolated yields. Only aryl iodides can be used for the reaction, because the use of the corresponding bromides leads to significantly lower isolated yields of the target compounds, **S20-2**. The developed reaction conditions can also be used for the *N*-arylation of imidazole, pyrrole, 7-azaindole, and indazole. Alternatively, 5 mol% of copper(II) sulphate has been used to catalyze indole and azole *N*-arylation in the presence of sodium hydroxide at 110 °C in dimethyl sulfoxide [92].

Copper nanocatalysts have become a popular tool for the preparation of *N*-arylindoles. Recent examples of the use of such nanocatalysts for the synthesis of *N*-arylindoles are listed in Table 1. In most cases, nanocatalysts catalyze the *N*-arylation of indoles under ligandless conditions. An exception to this concerns the preparation of *N*-arylindoles in the presence of 1,10-phenanthroline as a ligand, which is catalyzed by electrospun copper oxide nanoparticles [93] and ceria-supported copper [94] (Table 1, entries 1 and 2). Other copper-based nanocatalysts include CuO nanoflakes (CuO NFs@MP) prepared by reducing copper(II) chloride with fruit waste [95] and CuO nanoparticles, which are easily available by reducing copper(II) acetate by means of water extraction from fresh *Rosa canina* fruits [96] (Table 1, entries 3 and 4). The structures of both nanocatalysts were determined using physicochemical methods, including X-ray diffraction, Fourier transform infrared spectroscopy, field emission scanning electron microscopy, and others. However, the testing of their ability to catalyze the *N*-arylation of indoles is limited to only a few cases. Substantially better scopes were reported for carbon nanotube-copper oxide (CNT–CuO) [97], copper oxide nanoparticles [98,99], and copper nanoparticles decorated with organically modified montmorillonite (copper-decorated OMMT) [100] (Table 1, entries 5–7).

**Scheme 22:**
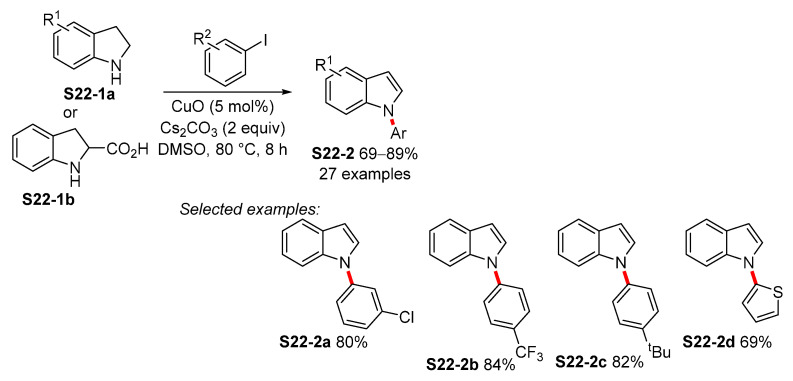
Synthesis of *N*-arylindoles from indolines or indoline carboxylic acids.

An alternative route to *N*-arylindoles is based on the two-step *N*-arylation of indoles starting from either indolines, **S22-1a**, or indoline carboxylic acids, **S22-1b** (Scheme 22) [101]. The reaction of the starting compounds **S22-1a** and **S22-1b** with predominantly aryl iodides is catalyzed by a recyclable nano-copper oxide catalyst. Based on the available literature, the authors assume that the preparation of the target compounds **S22-2** starts with the aromatization of indolines into indoles, followed by *N*-arylation. During this study, 27 *N*-arylated indoles were prepared. Selected examples **S22-2a**–**S22-2d** show that the scope of the reaction is mainly limited to unsubstituted indoles and aryl iodides with simple substituents. The authors also confirmed that the nano-copper oxide catalyst can be reused in up to four cycles without a significant loss in activity.

Copper catalysts and nitrogen ligands represent a popular combination for cross-coupling reactions. This is also true for the *N*-arylation of indoles. A first example of the use of *N*-ligands concerns the preparation of *N*-arylindoles, which is catalyzed by copper iodide and ligand **L1** (Table 2, entry 1) [102]. The authors succeeded in the preparation of 25 *N*-arylindoles, although a limited number of functional groups were introduced by this procedure. Significantly greater tolerance on the part of the functional groups was achieved by using the hydroxyquinalidine ligand **L2** (Table 1, entry 2) [103]. In contrast to previous work [102], the *N*-arylation can be performed at 90 °C, and the reaction can also be used to prepare *N*-aryl imidazoles, pyrazoles, pyrroles, benzoimidazoles, and carbazoles. Ligands in the form of natural amino acids allow the *N*-arylation of indoles under similarly mild reaction conditions [103]. Unfortunately, the scope of the reaction is almost exclusively limited to the preparation of *N*-arylindoles (Table 2, entries 3 [104] and 4 [105]). The use of metformin as the ligand for *N*-arylation led to the formation of *N*-arylindoles, although the reactions were carried out in DMF at 130 °C (Table 2, entry 5) [106]. The scope of this *N*-arylation is limited to the preparation of *N*-arylindoles.

**Scheme 23:**
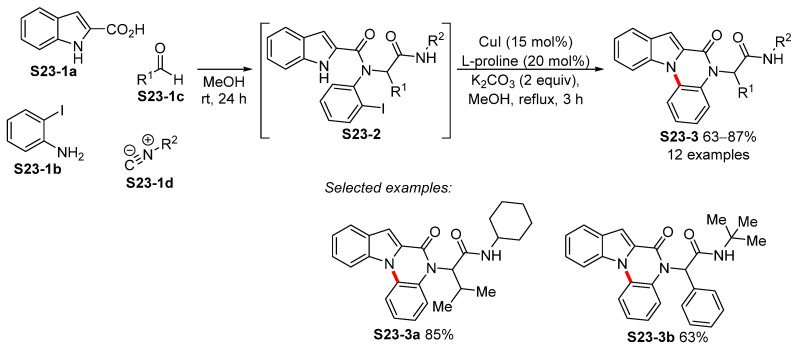
Ugi four-component reaction and subsequent post-modification for the synthesis of indolo[1,2-a]quinoxalines **S23-3**.

The copper-catalyzed *N*-arylation of indoles in the presence of an L-proline ligand was used to prepare indolo[1,2-a]quinoxalines (Scheme 23) [107]. The authors used the Ugi multicomponent reaction to prepare 2-substituted indoles **S23-2**, and the subsequent intramolecular cyclization gave the target compounds **S23-3**. The Ugi reaction is limited to unsubstituted 2-iodaniline and 2-indolyl carboxylic acid. From the examples of **S23-3a** and **S23-3b**, it is clear that the scope of the two-step one-pot protocol for the synthesis of indolo[1,2-a]quinoxalines **S23-3** is limited to aldehydes and isonitriles without functional groups. It is worth noting that the copper-catalyzed *N*-arylation gave better yields of *N*-arylated indoles **S23-3** than the palladium-catalyzed cyclization (Pd(OAc)_2_, BINAP, K_2_CO_3_, toluene, reflux, 24 h).

**Scheme 24:**
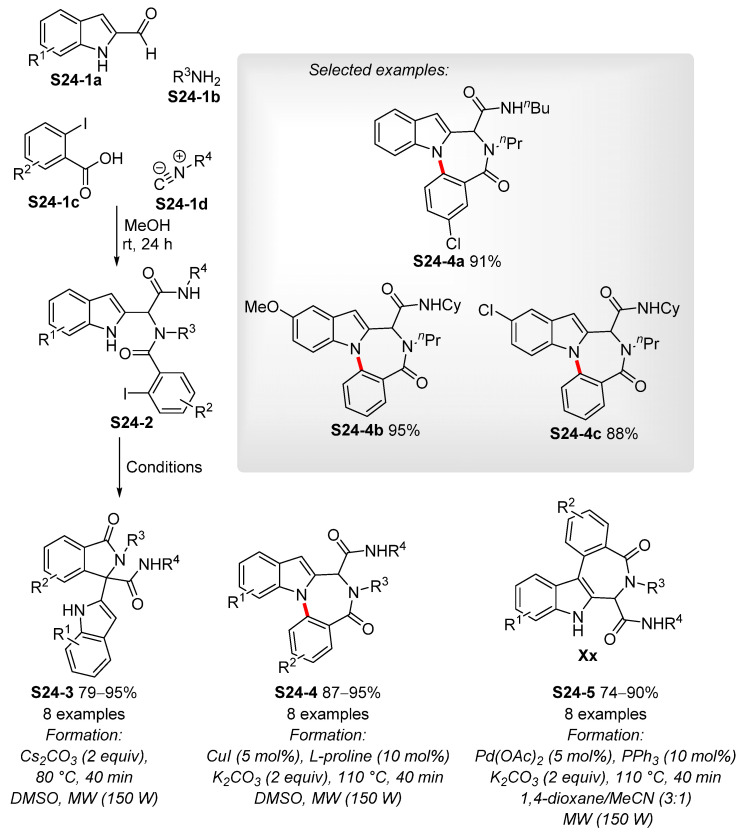
Diverse synthesis of heterocyclic compounds from the Ugi adducts **S24-2**.

A similar approach for the intramolecular cyclization of Ugi adducts was reported by Liu in 2013 (Scheme 24) [108]. In this approach, the Ugi reaction is carried out among 2-indolylcarbaldehyde **S24-1a**, 2-iodobenzoic acid **S24-1c**, amine **S24-1b**, and isonitriles **S24-1d**. The Ugi adduct **S24-2** is subsequently cyclized under different conditions. The *N*-arylation products **S24-4** are obtained via a copper-catalyzed reaction in the presence of an L-proline ligand. The products of 2,3-cyclization **S24-5** are formed during the palladium-catalyzed reaction. Cyclization performed without a transition metal produced isoindolin-1-ones **S24-3**. The scope of the discovered transformations is limited to a few examples, although a more extensive scope was reported by the same author in 2014 [109].

*N*,*N*′-Dimethylethylenediamine (DMEDA) is a popular ligand for use in the copper-catalyzed *N*-arylation of indoles. Lim used stoichiometric amounts of copper oxide for the arylation of indoles in pyridine (Table 3, entry 1) [110]. Unfortunately, the reaction scope is limited to the preparation of indoles with an ethoxycarbonyl group at position 2 of the indole moiety. An interesting route for the preparation of functionalized *N*-arylindoles was reported in 2013 (Table 3, entry 2) [111]. In their study, Ham and Kim developed the chemoselective arylation of halogen aryltrifluoroborates to form functionalized organotrifluoroborates. Only two indole derivatives were prepared by this procedure (in 72% and 73% yields, respectively). The presence of a trifluoroborate group in the structure of *N*-arylindoles can be used in the Suzuki reaction to prepare *N*-arylindoles with more complex substituents at position 1 [111]. The complementary method for the *N*-arylation of indoles was performed under similar reaction conditions (Table 3, entries 3 and 4) [112,113].

**Scheme 25:**
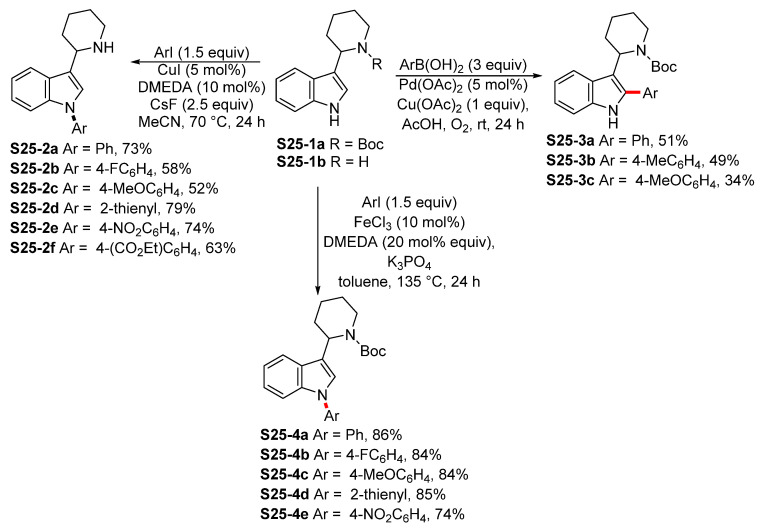
Dichotomies in the transition-metal-catalyzed arylation of substituted indoles.

Dichotomies during the arylations of 3-substituted indoles **S25-1a** and **S25-1b** were reported by Schnürch (Scheme 25) [114]. The starting indole, which featured a Boc-protecting group, reacted with arylboronic acids in the presence of a palladium catalyst and one equivalent of copper acetate to give 2-arylindoles **S25-3a**–**S25-3c** in moderate yields. The formation of *N*-arylindoles was not observed in this case. The low yields of indoles **S25-3a**, **S25-3b**, and **S25-3c** were explained by the inferior steric requirements for substitution at position 2. Changing the catalytic system to ferric chloride with a DMEDA ligand led to the formation of *N*-arylated indoles **S25-4a**–**S25-4e** in high yields. *N*-arylation can also be performed in the case of unprotected indole **S25-1b**, although in this case a catalytic amount of copper iodide with a DMEDA ligand and cesium fluoride as a base had to be used.

A series of *N*-arylation reactions using ligands or reagents obtained from natural sources included the *N*-arylation of indoles, which was carried out in glycerol (Table 4, entry 1) [115]. Glycerol is considered a cheap and environmentally-friendly alternative to the commonly used solvents DMF and DMSO. A common feature of *N*-arylation reactions performed in glycerol, DMF, and DMSO is the high reaction temperature. The authors hypothesize that one equivalent of DMSO acts as a ligand to help the oxidative addition of the copper catalyst to the C-halogen bond. The good solubility of the reaction products in organic ethers allowed the recycling of the catalytic system dissolved in glycerol by extraction of the products and the starting compound to the ether. Such recycled catalysts can be used in three catalytic runs. A different ligand concept for the *N*-arylation of indoles makes use of methyl-α-d-glucopyranoside **L1** and glucosamine **L2** as ligands for copper-catalyzed *N*-arylations, as reported by Xuan [116] and Chen [117] (Table 4, entries 2 and 3). Neither reaction is significantly different from the other procedures available for the *N*-arylation of indoles. In the case of the glucosamine ligand **L2**, the formation of a radical intermediate **T4-1** was proposed, although the formation of this intermediate has not been experimentally verified. The use of other natural substances in this regard includes the use of *N*-alkyl-glucosamine as a sugar-based surfactant [118], alpha-d-galacturonic acid **L3** [119], and l-(-)-quebrachitol **L4** [120] as ligands for the copper-catalyzed *N*-arylation of indoles and nitrogen-containing substances.

In addition to the above-mentioned examples, other ligands containing a nitrogen atom represent popular choices for the copper-catalyzed arylation of indoles. Nitrogen ligands also form part of the system for the *N*-arylation of indoles in water (Table 5). Schmitt used copper bromide and the ligand **L1** for the *N*-arylation of indoles under mild reaction conditions (Table 5, entry 1) [121]. d-Glucose was used as the reducing agent in this case. The reaction was performed in a commercially available dl-α-tocopherol methoxypolyethylene glycol succinate solution. It was experimentally verified that the use of a surfactant is necessary to achieve a high yield of *N*-arylindoles. The developed reaction conditions were used for the synthesis of 11 *N*-arylindoles, and the tolerance of the functional groups in this case was limited to halogens, nitrile, ethers, and the formyl group. A similar surfactant-based strategy for preparing *N*-arylindoles was reported by Liu in 2013 (Table 5, entry 2) [122]. The reaction conditions for the *N*-arylation of indoles make use of the bipyridine ligand **L2**, potassium phosphate as the base, and betaine as the surfactant. In contrast to previous work [121], the arylation reaction must be performed at 90 °C. The authors demonstrated the tolerance of the acetyl group during the preparation of ten indole derivatives under optimized reaction conditions. An extensive series of *N*-arylated indoles was prepared by means of indole arylation catalyzed by copper iodide and the phenanthroline ligand **L3** in 30% aqueous 1,2-dimethoxyethane (DME) (Table 5, entry 3) [123]. The optimum ratio of DME to water was determined using kinetic experiments, which showed that reactions performed in pure water or DME did not achieve quantitative conversion. An alternative procedure for the preparation of *N*-arylindoles relies on the reaction of boronic acids with indoles in aqueous ammonia and potassium *tert*-butoxide as a base under ligandless conditions [124]. To complete the list of *N*-arylations of indoles in water, it is necessary to mention the *N*-arylation procedures associated with heterocyclic compounds and amines, which can be applied for the preparation of a limited number of *N*-arylindoles [125,126,127,128,129,130,131].

The copper(I)-oxide-catalyzed *N*-arylation of indoles, as catalyzed using copper oxide and a pyridine *N*-oxide ligand **L1**, was reported in 2016 (Table 6, entry 1) [132]. By varying the ligands during the optimization of the reaction conditions, it was confirmed that the presence of both pyridine *N*-oxide and phenyl amide moieties in the structure of the ligand **L1** is necessary to achieve maximum yields. The optimized reaction conditions are suitable for the preparation of *N*-aryl pyrazole, pyrrole, and indazole. The reaction scope for indoles is limited to six examples. The use of a pyridine *N*-oxide ligand was presumably inspired by prior studies that utilized pyridine *N*-oxide ligands for general *N*-arylation [133,134,135]. The pyridine-based ligand **L2**, which contains an iminopyridyl moiety, was used for the *N*-arylation of indoles in DMSO at 120 °C (Table 6, entry 2) [136]. Another ligand **L3** that contains an iminopyridyl moiety in its molecule was reported in 2021 (Table 6, entry 3) [137]. A short communication on the copper-catalyzed *N*-arylation of indoles was published in 2015 (Table 6, entry 4). Copper(I) chloride with a phosphine ligand **L4** in DMSO was found to be the best combination for the *N*-arylation of indoles. The scope of the reaction is limited to aryl halides with electron-donating or electron-neutral substituents. It should be noted that 4-iodobenzonitrile did not react under the optimized reaction conditions.

**Scheme 26:**
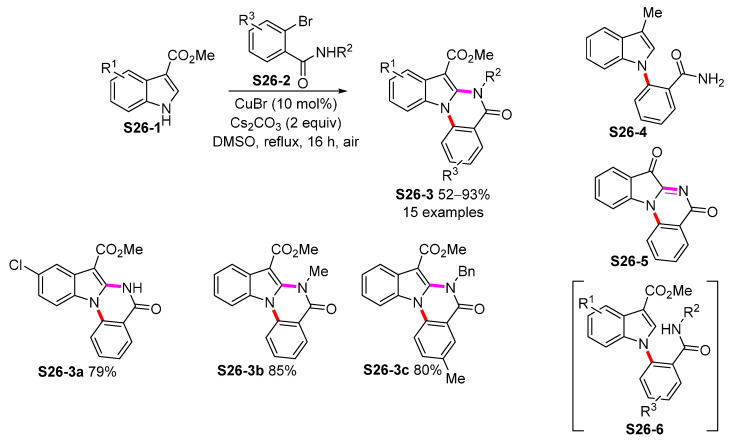
Formal double *N*-arylation synthesis of indolo[1,2-a]quinazolinones **S26-3** via self-relay copper catalysis.

Recently, a self-relaying copper(I)-catalyzed sequence was used for the preparation of indolo[1,2-a]quinazolinones **S26-3** by means of the reaction of the methyl esters of 3-indolylcarboxylic acid **S26-1** with 2-bromobenzamides **S26-2** (Scheme 26) [138]. This reaction is sensitive to the structure of the starting indole derivative, as the use of the 3-methylindole led to the formation of the *N*-arylated indole **S26-4** and indoloquinazolinone **S26-5**. Fifteen indolo[1,2-a]quinazolinone **S26-3** derivatives were prepared using this procedure. It is worth noting that despite the harsh reaction conditions, the presence of a chlorine atom was tolerated. It was also verified that the *N*-arylation of indoles to form the intermediate **S26-6** is the first step in the formation of the target compounds **S26-3**.

**Scheme 27:**
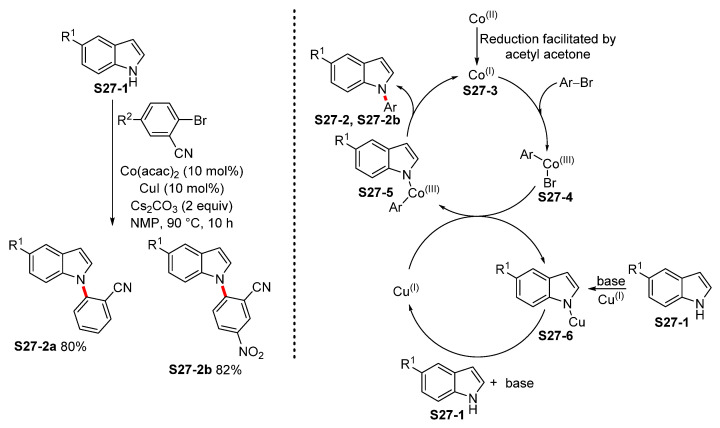
Cobalt–copper-cocatalyzed *N*-arylation of indoles.

The cobalt–copper-cocatalyzed *N*-arylation and alkenylation of *N*-heterocycles was reported by Ran (Scheme 27) [139]. The reaction conditions were optimized for the alkenylation of amides, although it was determined that these conditions could also be used for the preparation of *N*-arylindoles **S27-2a** and **S27-2b** in high yields. The authors assume that in the first step, the Co^(II)^ is reduced to Co^(I)^ by means of acetylacetone. The oxidative addition is followed by the substitution of the halide ligand, which is facilitated by the formation of the Cu(I)–N complex **S27-6**. The product of the reaction is then obtained by reductive elimination. The proposed mechanism was partially confirmed experimentally. A somewhat different approach to the *N*-arylation of nitrogen-containing heterocycles makes use of the iron-catalyzed bromination of aromatics, followed by copper-catalyzed *N*-arylation [140]. This approach was used for the synthesis of 1-(4-methoxyphenyl)indole in a 78% yield.

**Scheme 28:**
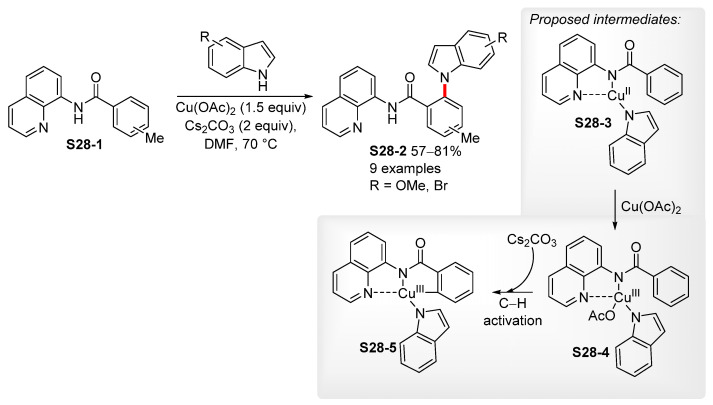
C–H activation for the *N*-arylation of indoles.

The general copper-catalyzed *N*-arylation of indoles and other azoles based on C–H bond activation was reported in 2016 (Scheme 28) [141]. The developed methodology is based on *N*-(quinolin-8-yl)benzamides **S28-1**, which react with azoles and indoles to give *ortho*-substituted benzamides **S28-2**. The methodology was used for the preparation of *N*-aryl pyrroles, indoles, carbazoles, and pyrazoles. The presence of a quinolin-8-yl group facilitates the formation of the complex **S28-3** by means of copper coordination to the quinolin-8-yl moiety. The oxidation of Cu^II^ to Cu^III^ forms complex **S28-4**, which undergoes intramolecular C–H cupration. Eight *N*-arylated indoles were prepared using this procedure. It was shown that benzamide with a pyrrolyl unit can be hydrolyzed into *ortho*-substituted benzoic acid. The same research group showed that the methodology developed for C–H functionalization reactions can be extended to picolinamides, rather than benzamides [142].

**Scheme 29:**
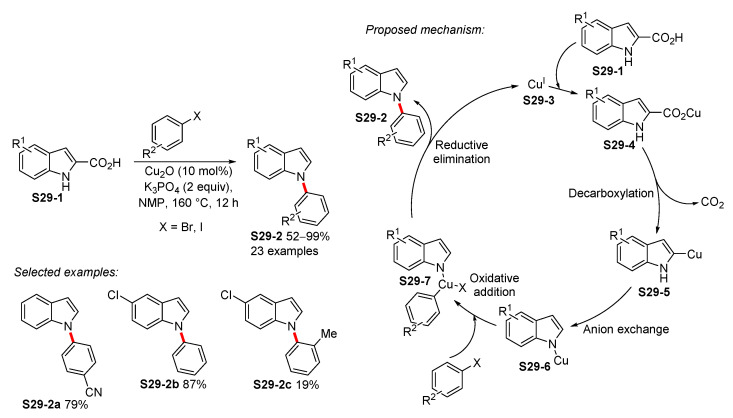
Decarboxylative *N*-arylation of 2-indolylcarboxylic acids.

An alternative approach to the *N*-arylation of indoles makes use of 2-indolylboronic acids (Scheme 29) [143]. The starting carboxylic acids **S29-1** react with aryl bromides or aryl iodides in the presence of catalytic amounts of copper oxide and potassium phosphate as a base. The reported scope of the reaction is limited to substituted phenyl iodides and bromides. Other aromatic and heteroaromatic halides were not tested. In terms of the proposed mechanism, the authors assume the formation of copper carboxylate **S29-4** and the subsequent decarboxylation into the 2-indolyl copper reagent **S29-5**. The final products **S29-2** are formed via anion exchange, oxidative addition, and reductive elimination steps.

**Scheme 30:**
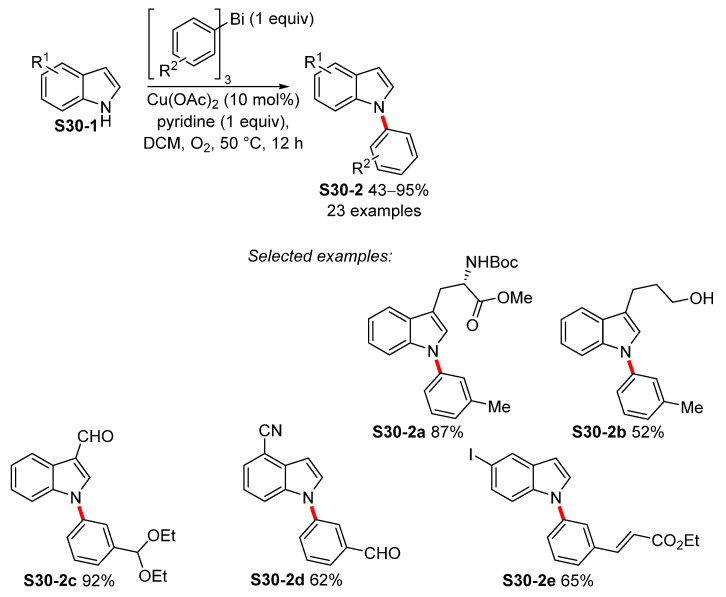
*N*-arylation of indoles by means of triarylbismuth reagents.

Arguably the most extensive procedure for the preparation of *N*-arylindoles is based on the reaction of indoles with triarylbismuth reagents (Scheme 30) [144]. The reaction is catalyzed by copper acetate in the presence of oxygen and one equivalent of base. The triarylbismuth reagents can be prepared using the reaction of bismuth(III) trichloride with Grignard reagents or by the chemical transformation of the ester or formyl group of the corresponding triarylbismuth reagents. The procedure is suitable for the preparation of highly functionalized indoles because the methodology can tolerate a variety of functional groups, including a formyl or ester group, as illustrated by selected examples **S30-2b**–**S30-2e**. The tryptophan derivative **S30-2a** was also prepared using this procedure. In addition, a series of heteroatom arylations by organobismuth reagents has been reported [145,146,147,148].

**Scheme 31:**
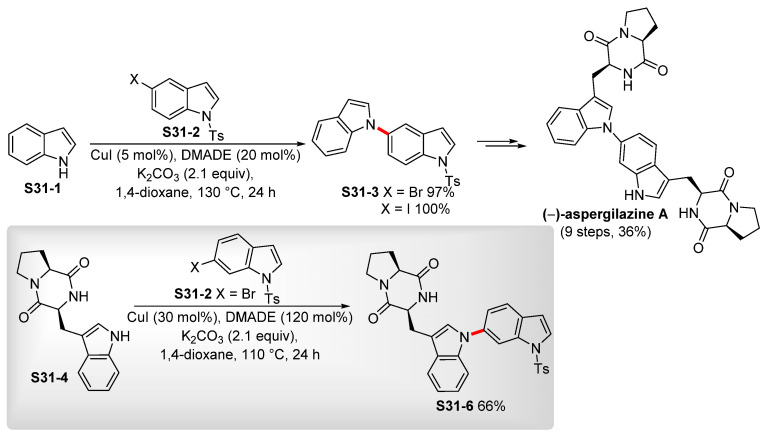
Total synthesis of (–)-aspergilazine A via the copper-catalyzed *N*-arylation of indoles.

An alternative approach for the preparation of (–)-aspergilazine A that uses copper-catalyzed indole *N*-arylation was reported in 2017 (Scheme 31) [149]. A direct approach to (–)-aspergilazine A starting from the tryptophan derivative **S31-4** proved inefficient because the expected product of the reaction **S31-5** was obtained in only a 66% yield. Thus, the preparation of the target compound from indole **S31-1** and 2-bromoindole or 2-iodoindole **S31-2** was accomplished in nine steps in a total isolated yield of 36%.

**Scheme 32:**
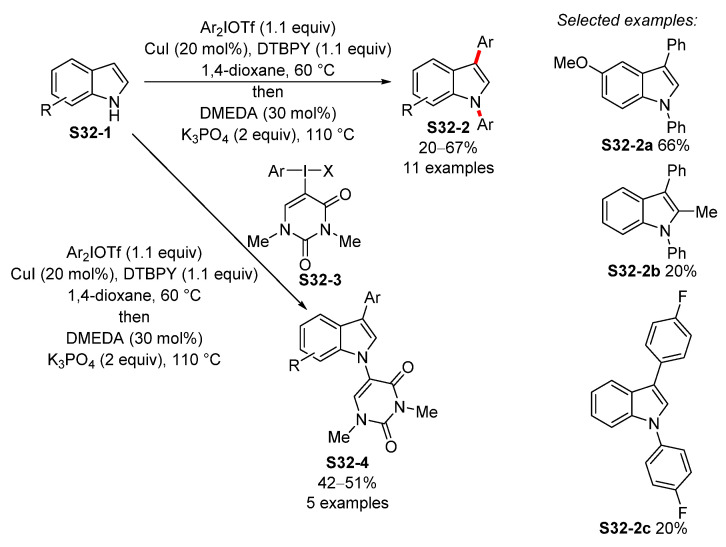
Atom-economical *N*-arylation of indoles by diaryliodonium salts.

Atom-economic arylation of indoles was described in 2015 (Scheme 32) [150]. N1-Unsubstituted indoles react with diaryliodonium salts in the presence of a catalytic amount of copper iodide to give N1- and C3-arylated indoles in average isolated yields. High selectivity was observed for aryl-uracil iodonium triflates **S32-3**, affording the 3-aryl-1-indoles-uracil conjugates **S32-4**.

**Scheme 33:**
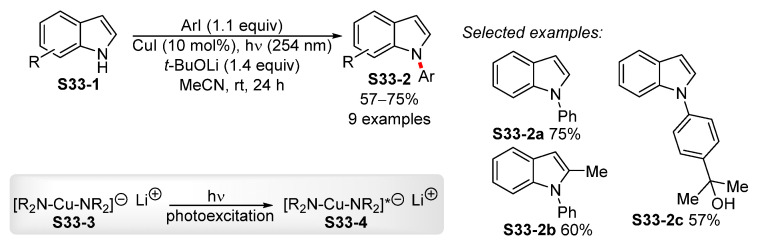
*N*-Arylation of indoles under mild conditions.

The combination of photocatalysis and copper-catalyzed *N*-arylation enabled the *N*-arylation of indoles at room temperature (Scheme 33) [151]. The key step in this conversion is the photoexcitation of a copper complex **S33-3**. The reaction is also suitable for the preparation of *N*-aryl benzimidazoles, imidazoles, and carbazoles.

### 3.4. Miscellaneous Transition-Metal-Catalyzed N–Arylation of Indoles

**Scheme 34:**
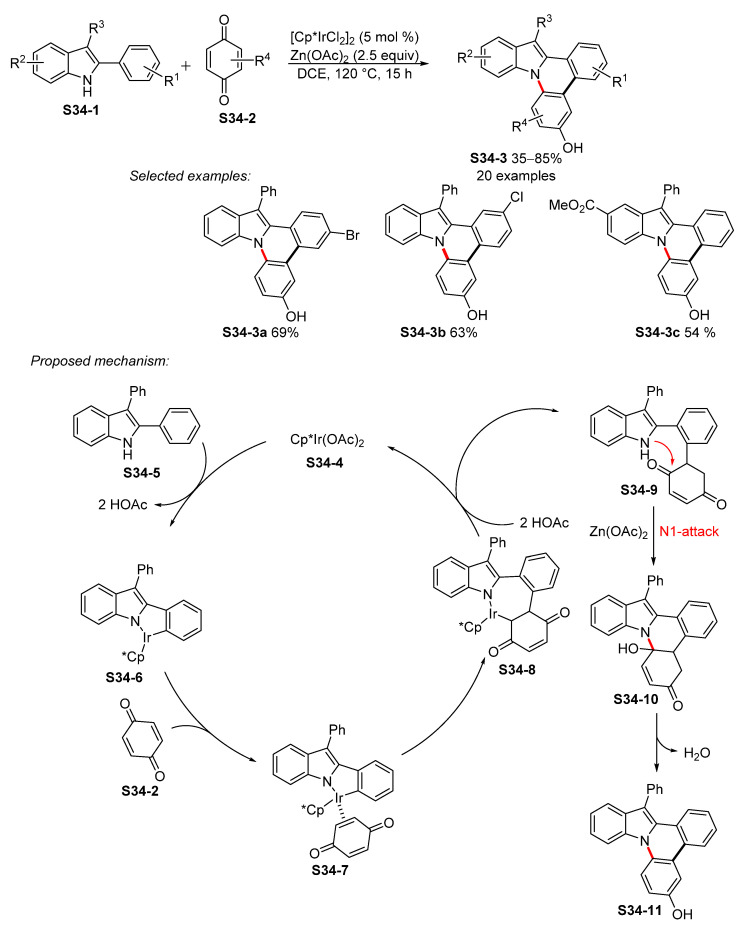
Iridium-catalyzed oxidative annulation of 2-arylindoles **S34-1**.

A straightforward approach to the preparation of indolo[1,2-f]phenanthridine derivatives **S34-3** based on the *N*–arylation of 2-aryl-3-substituted indoles **S34-1** using an iridium-catalyzed oxidative [4 + 2] annulation reaction with quinones **S34-2** was reported by Guo and Fan (Scheme 34) [152]. A series of 20 *N*-arylated indoles was prepared, all in satisfactory yields, under optimized reaction conditions. The indoles substituted at positions 2 and 3 smoothly reacted under optimized reaction conditions. However, the reaction is limited for benzoquinone and 2-methylbenzoquinone. Other quinone derivatives, such as 2-chlorobenzoquinone, 2,5-dimethylbenzoquinone, and naphthoquinone, did not furnish the corresponding products. The authors posited a plausible reaction mechanism on the basis of mechanistic studies. Initially, the iridium-catalyzed dual N–H/C–H bond activation of **S34-5** is responsible for the formation of five-membered iridacycle **S34-6**. The subsequent coordination of benzoquinone to the iridium complex **S34-6** affords the new iridacycle **S34-7**, which then undergoes a migratory insertion to deliver a seven-membered intermediate **S34-8**. Next, the protonolysis of the complex **S34-8** with HOAc leads to both intermediate **S34-9** and the active Ir(III) catalyst **S34-4**. Finally, a Zn(OAc)_2_-promoted intramolecular indolyl N1-attack on the carbonyl group affords the key intermediate **S34-10**, which undergoes a dehydration reaction to deliver the final product **S34-11**.

**Scheme 35:**
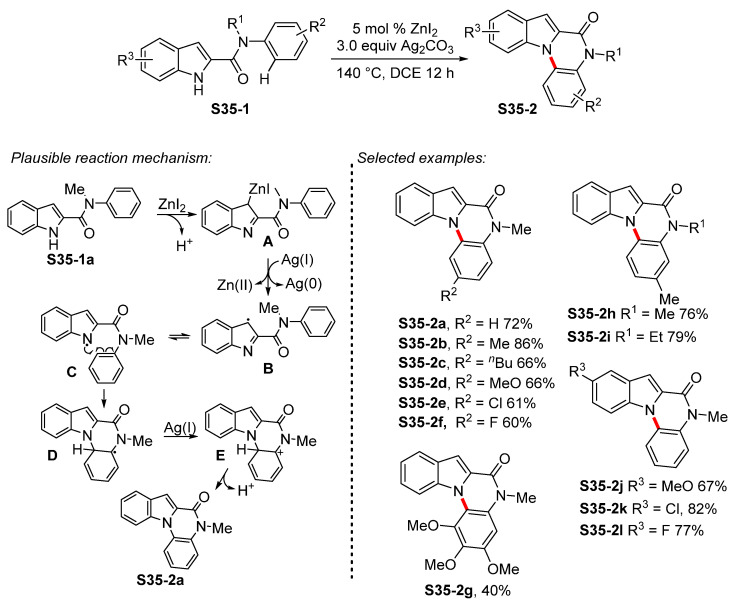
Novel method for the N–H arylation of indole-2-carboxamides **S35-1** developed.

An alternative approach to the *N*-arylation of indole-2-carboxamides was developed by Kong (Scheme 35) [153]. In this reaction, zinc(II) iodide is used as a catalyst and Ag_2_CO_3_ as an oxidant en route to indolo[1,2-a]quinoxalin-6-on **S35-2**. The scope of the reaction is limited to substrates with a methoxy group or halogens. The presence of the N–CH_3_ amide moiety in the starting compound is crucial for the success of this reaction, as no reaction occurred in the case of a substrate bearing an N−H amide group or a −CO_2_(4-MePh) group. It was proposed that this behavior is caused by the steric effect of the N–CH_3_ group, which can bring the two reaction sites closer, thereby facilitating the intramolecular cyclization reaction. A plausible mechanism can be identified on the basis of controlled experiments. The reaction is initiated by the generation of the indolylzinc(II) intermediate **A**. The oxidation of **A** by Ag_2_CO_3_ delivers the resonance-stabilized radical intermediate **B** or **C**. The intermediate **D** is obtained via the intramolecular addition of an indole radical onto the *N*-aryl moiety, which is followed by oxidation and deprotonation to give the desired product **S33-2a**.

## 4. *N*-Arylated Indoles as Biologically Active Substances

**Figure 5:**
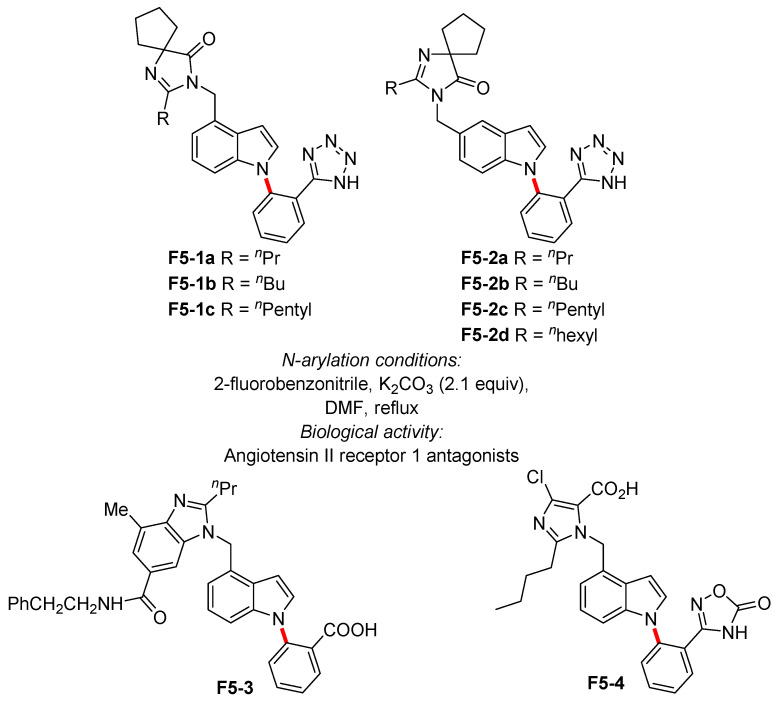
Selected examples of bioactive *N*-arylindoles—part 1.

**Figure 6:**
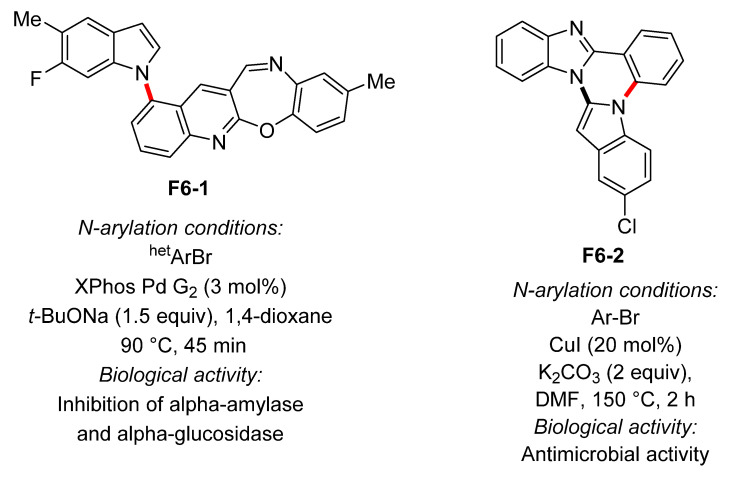
Selected examples of bioactive *N*-arylindoles—part 2.

**Figure 7:**
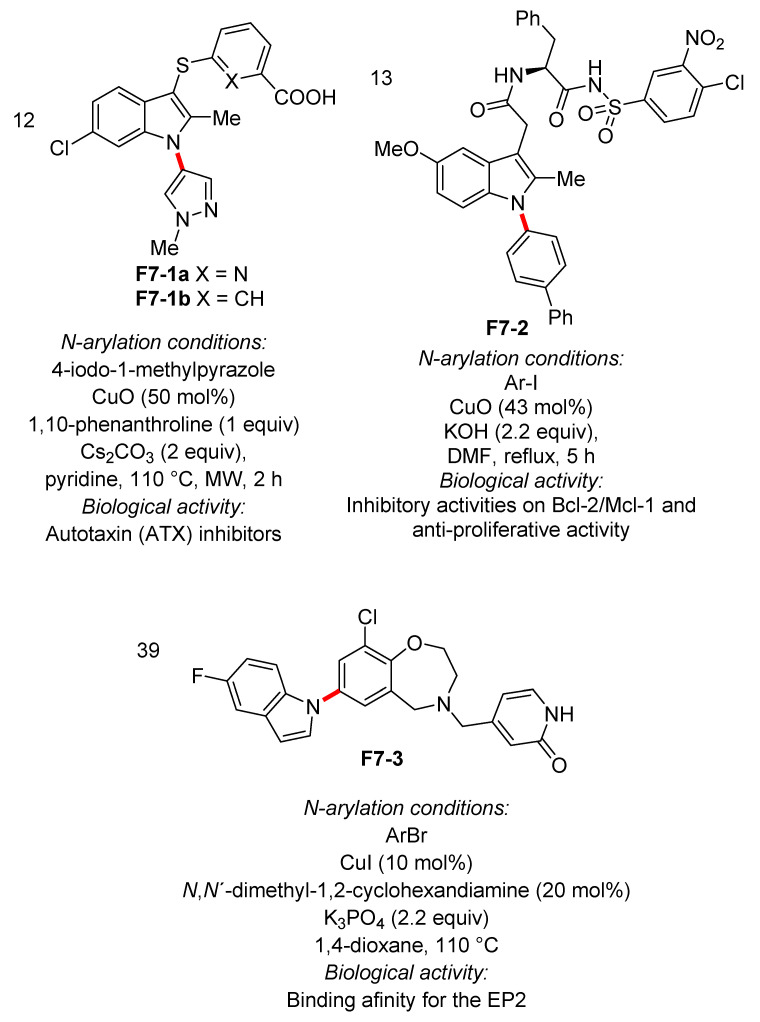
Selected examples of bioactive *N*-arylindoles—part 3.

**Figure 8:**
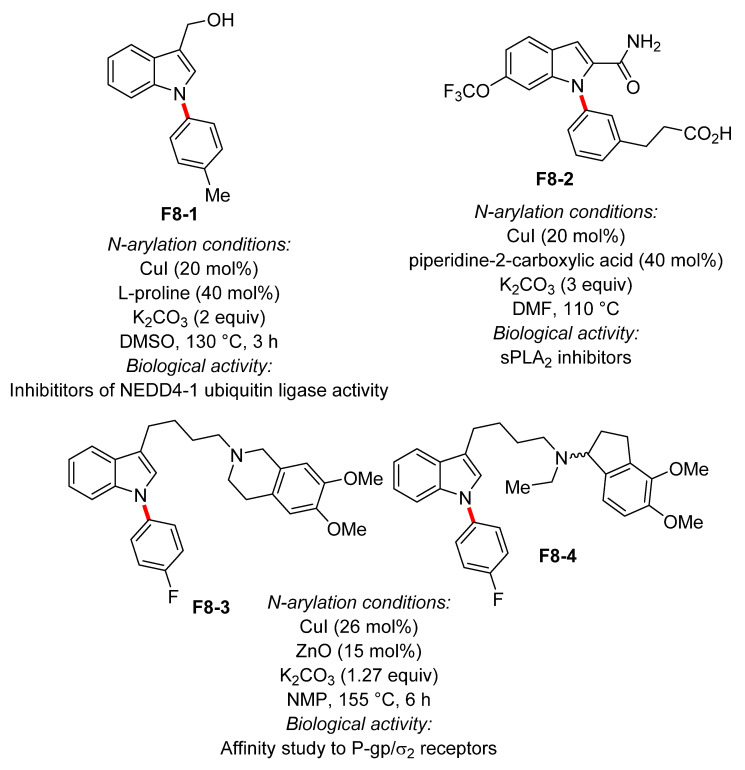
Selected examples of bioactive *N*-arylindoles—part 4.

**Figure 9:**
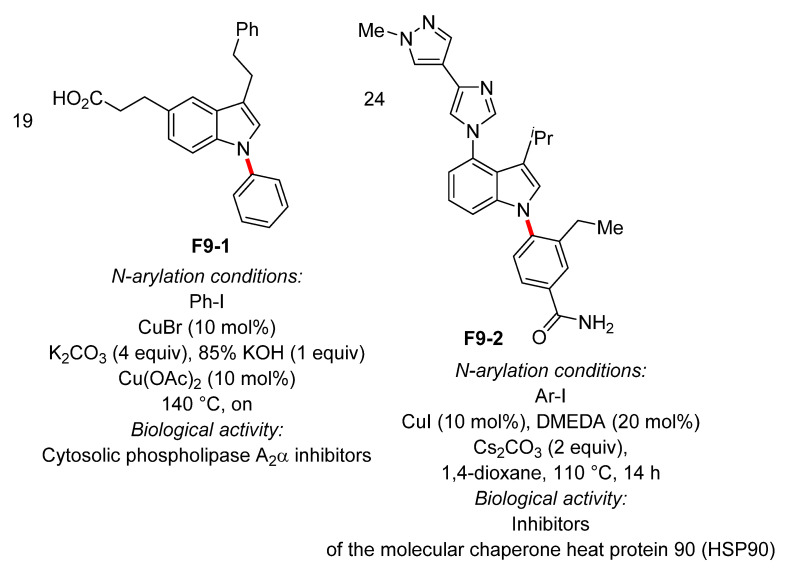
Selected examples of bioactive *N*-arylindoles—part 5.

In the preceding sections, we have discussed a wide variety of procedures that can be used for the preparation of *N*-indoles. Such a large number of different procedures have been developed due to the near endless number of applications of *N*-arylindoles. Among them, *N*-arylindoles have found applications in the field of materials chemistry. For example, *N*-arylindoles have been proposed as novel dye-sensitized solar cells [154], electrophosphorescent diodes [155], oxygen sensors [156], and other things [157,158,159,160]. Procedures for the *N*-arylation of indoles have also been used for the preparation of indoles with biological activity. A number of *N*-arylindoles have been prepared in structure–activity relationship (SAR) studies in an effort to verify the mechanisms of the biological activity of the substituted indoles; and the determination of the biological activities of *N*-arylindoles has also been part of a large-scope synthetic study concerning the preparation of *N*-arylindoles. Examples of such works include inhibitors of human neutrophil elastase (HNE) [161], centromere-associated protein-E (CENP-E) inhibitors [162], CRTh2 antagonists [163], and determinations of cytotoxic activity [164,165]. Ultimately, a large group of *N*-arylindoles was prepared to study their biological activity. Selected examples are given in Figure 5, Figure 6, Figure 7, Figure 8 and Figure 9.

A series of indole derivatives, **F5-1a–F5-1c** and **F5-2a–F5-2d**, was prepared to serve as angiotensin II (Ang II) receptor 1 antagonists (Figure 5) [166]. The introduction of the aryl substituent was accomplished through nucleophilic aromatic substitution by means of the reaction of substituted indoles with 2-fluorobenzonitrile in the presence of potassium carbonate in refluxing DMF. Among the prepared *N*-arylindoles, the compound **F5-2b** showed binding affinity to the angiotensin AT_1_ receptor, with an IC_50_ value of 1.26 ± 0.08 nM in radioligand binding assays. The same compound also showed the strongest antihypertensive effect, with a maximum reducing response of lower than 40.62 ± 4.08 mmHg MBP at 15 mg/kg 4 h after oral administration in SHRs. The acute toxicity of compound **F5-2b** was determined by LD_50_ as 1551.71 mg/kg. Other indole derivatives have been prepared by the same group and studied as angiotensin II receptor 1 antagonists [167,168,169,170]. The examples given in Figure 5 show that a common feature of these compounds is the presence of an *ortho*-substituted phenyl ring with a carboxylic acid **F5-3** [167,168,170] or 1,2,4-oxadiazole **F5-4** [169] scaffold at position 1 of the indole unit. Similarly, the introduction of phenyl substituents was accomplished via nucleophilic aromatic substitution in refluxing DMF.

The new quinoline derivatives were prepared by means of a Buchwald–Hartwig reaction, which was catalyzed using a XPhos Pd G2 catalyst and *^t^*BuONa as the base (Figure 6) [171]. A number of compounds, including the indole derivative **F6-1**, were prepared using this procedure. It was determined that the substance **F6-1** inhibited 70% of the α-amylase at a concentration of 50 μg/mL, and it also inhibited α-glucosidase at a concentration of 50 μg/mL. Benzimidazole and indole conjugates were prepared via double copper-catalyzed C–N bond formation [172]. The selected compound **F6-2** demonstrated the formation of a N^indole^–C^phenyl^ bond by a copper(I)-iodide-catalyzed reaction in DMF at 150 °C. The substance **F6-2** showed the best minimum inhibitory concentration (MIC) of 4 μg/mL against Gram-negative (*E. coli*, *P. putida*, and *S. typhi*) and Gram-positive (*B. subtilis* and *S. aureus*) bacteria. The antifungal activity against *C. albicans* and *A. niger* of **F6-2** is represented by an MIC value of 16 μg/mL.

A number of indole derivatives with a 1-methylpyrazol-4-yl substituent at position 1 were investigated with respect to their inhibitory activity in relation to the autotaxin (ATX) enzyme, which is responsible for the hydrolysis of lysophosphatidylcholine (LPC) (Figure 7) [173]. The introduction of the pyrazole skeleton was performed by means of Ullman condensation with copper oxide and 1,10-phenanthroline ligand in pyridine at 110 °C under microwave conditions. A detailed SAR study showed that the indole derivative **F7-1a** is a potent compound with an IC_50_ value of 22 ± 6.9 nM in the Bis-pNPP assay and 4 ± 0.5 nM IC_50_ in the LPC assay. Similar results were observed for the benzoic derivative **F7-1b**. The crystal structure of the studied compounds indicated that the active compounds bind within the tunnel, which was described as an allosteric binding site. Both B-cell lymphoma (Bcl-2) and myeloid cell leukemia sequence 1 (Mcl-1) are proteins that help with the development and survival of multiple tumor types. Therefore, Fang [174] focused on studying the properties of indole derivatives with a phenyl substituent at position 1 as compounds that exhibit potent inhibitory activity against Bcl-2. The introduction of the aryl substituents was again catalyzed by cuprous oxide, although the reactions were performed in refluxing DMF. Compound **F7-2** showed the best activity against Bcl-2, with a K*i* value of 0.35 ± 0.03 μM. Docking studies revealed that the active compound **F7-2** binds to the active pocket of the Bcl-2 protein through a hydrogen bond with Arg143 and the π–π interaction with Ph109 and Phe101. A number of 2,3-dihydrobenzo[f][1,4]oxazepine derivatives were prepared as antagonists of the prostaglandin E2 receptor subtype 2 (EP2) [175]. The oxazepine derivative with a 4-fluoroindole substituent **F7-3** showed a binding affinity of 8 nM for the hEP2 receptor and an IC_50_ value of 50 nM in the hEP2 cAMP assay. In addition, the compound lacked CYP inhibition at 3 μM and had a 4000-fold higher binding affinity for the hEP2 receptor when compared with hEP1, hEP3, and hEP4. The formation of a N–C^aryl^ bond was accomplished via a CuI-catalyzed reaction in the presence of a *N*,*N*′-dimethyl-1,2-cyclohexandiamine ligand and potassium phosphate as the base in 1,4-dioxane at 110 °C.

New substituted indole-3-carbinoles, as potent anti-cancer compounds derived from indole-3-carbinol (I3C), were designed and prepared as new inhibitors of NEDD4-1 ubiquitin ligase activity (Figure 8) [176]. In this case, the *N*-arylation of indoles was accomplished by means of an l-proline ligand and a copper catalyst. The parent carbinol I3C reached an IC_50_ value of 284 μM. The introduction of a benzyl group into position 1 decreased the IC_50_ value to 12.3 μM, whereas the introduction of the 4-tolyl group **F8-1** substantially improved the IC_50_ value to 2.71 μM. Protein thermal shift assays in combination with in-silico binding simulation revealed that the tested derivatives bind to the purified catalytic HECT domain of NEDD4-1. In 2018, Knerr published two papers that evaluated *N*-arylindoles with trifluoromethoxy and carboxamide groups as selective inhibitors of secreted phospholipase A_2_ type X [177,178]. The formation of an N–C^aryl^ bond was achieved by means of copper-catalyzed Ullman condensation in the presence of piperidine-2-carboxylic acid as a ligand. The indole **F8-2** exhibited the best IC_50_ value (0.022 μM) against sPLA_2_-X. The presence of a trifluoromethoxy group is the key to achieving high inhibitory activity. The substitution of CF_3_O for the methoxy group led to the IC_50_ value being reduced to 0.62 μM. In another two papers [179,180], the selective activity of *N*-arylindoles toward P-glycoprotein (P-gp) or σ_2_ receptor agents was studied. The indole derivative **F8-3** was chosen as a good ligand for both P-glycoprotein (P-gp) and the σ_2_ receptor. The starting compound **F8-3** was subject to SAR studies wherein the *N*-arylation of indoles was performed by a copper-catalyzed reaction in the presence of zinc oxide. On the basis of these studies, the compound **F8-4** as a (+)-(S)-enantiomer displayed notable nanomolar σ_2_ affinity [180].

Another set of indole derivatives with a similar substitution at position 1 of the indole skeleton was reported by Tamoo [181] and Uno (Figure 9) [182]. On the basis of extensive SAR studies, it was found that **F9-1** is an inhibitor of cPLA_2α_ (with an IC_50_ value of 0.075 μM) [181], and indole **F9-2** is a selective inhibitor of both HSP90 (IC_50_ = 0.069 μM) and SK-Br-3 (IC_50_ = 0.33 μM) [182]. The introduction of the aryl substituents was accomplished via copper-catalyzed Ullman condensation at reaction temperatures above 100 °C.

## 5. Conclusions

This review article has summarized the current state-of-the-art procedures for the preparation of *N*-arylindoles. The syntheses of the target compounds can be accomplished in several ways. The simplest procedures are based on transition-metal-free reactions. The second approach makes use of transition-metal-catalyzed aminations of organohalogenides or organometallic compounds. The metal most commonly used for the *N*-arylation of indoles is arguably copper, although a number of processes use palladium and nickel catalysts. The *N*-arylation of indoles can be performed in an inter- or intramolecular manner in high isolated yields. Despite considerable efforts having been made to successfully accomplish *N*-arylation experiments, two major limitations persist. The first limitation is associated with the low tolerance of the functional groups. Only the *N*-arylation of indoles, which is accomplished by the effect of triorganobismuth reagents, avoids this trend. The second limitation concerns the reaction temperatures at which the *N*-arylations take place. In the vast majority of cases, the *N*-arylations are performed at high temperatures, which commonly exceed 100 °C. In some cases, the arylation reactions are also performed in refluxing DMF. However, *N*-arylations of indoles at room temperature are rare. Thus, the development of new catalytic systems for the *N*-arylation of indoles represents a new direction of development in this field. Such efforts will result in higher tolerance of the functional groups, which should streamline the development of new biologically active compounds based on substituted indoles. At the same time, efforts to lower the reaction temperature required for the *N*-arylation of indoles will greatly benefit the overall economic aspects of the process.

## Figures and Tables

**Table 1:** Nanocatalyzed *N*-arylations of indoles.

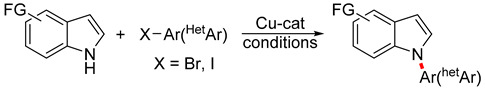				
Entry	Conditions	No. of Examples	^indole^FG	^Ar/hetAr^FG
1	CuO nano (5 mol%), 1,10-phen (50 mol%) KOH (2 equiv), DMSO, reflux, 24 h	4 56–80%	–	Cl
2	Cu@CeO_2_ (5 mol%), 1,10-phen (50 mol%) KOH (2 equiv), DMSO, reflux, 24 h	6 82–89%	–	Cl
3	PhI, CuO NFs@MP (15 mg/mmol) K_2_CO_3_ (1.5 equiv), DMF, 100 °C, 6 h	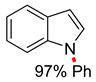		
4	CuO NPs (10 mg/1 mmol of indole), TEA (2 equiv), DMF, reflux, 2 h	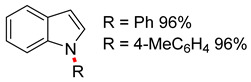		
5	CNT–CuO (5 mol%), *^t^*BuONa (3 equiv), DMSO, 120 °C, 12 h	21 42–95%	Cl, F	Cl, F, COMe, CO_2_Me, CN, CF_3_, 3,4-(CH_2_OCH_2_), NO_2_, SMe
6	CuO nano (5 mol%), K_2_CO_3_ (1 equiv), DMF, reflux, 8 h	15 62–98%	Br, CN, NO_2_, OMe	NO_2_
7	Copper-decorated OMMT (5 mol%), K_2_CO_3_ (2 equiv), DMSO, 130 °C, 6 h	8 73–97%	CN	OH, NH_2_,

**Table 2:** Nitrogen-based ligands for the *N*-arylation of indoles.

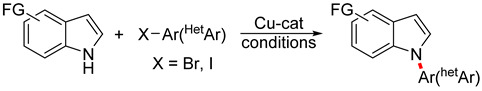					
Entry	Conditions	No. of Examples	^indole^FG	^Ar/hetAr^FG	
1	CuI (15 mol%)/**L1** (15 mol%) *^t^*BuOK (2 equiv), DMSO, 120 °C, 36–48 h	25 50–93%	CH=O, OMe	NO_2_, OMe,	
2	CuI (2 mol%)/**L2** (4 mol%) K_2_CO_3_ (2.5 equiv), DMSO, 90 °C	19 31–98%	–	Cl, CF_3_, CN, COCH_3_, CO_2_H, F, NH_2_, NO_2_	
3	CuI (5 mol%)/L-proline (10 mol%), K_2_CO_3_ (5 equiv) DMSO, 80–90 °C, 24 h	18 51–97%	Br, Cl, F, MeO,	Br, Cl, F, OMe, OH	
4	CuI (5 mol%), L-methionine (10 mol%), K_2_CO_3_ (2 equiv), DMF, 100 °C, 24 h	16 72–92%	Br, F, NO_2_, OBn, OMe,	F, CF_3_, NO_2_,	
5	CuI (10 mol%), **L3** (20 mol%), Cs_2_CO_3_ (2 equiv), DMF, 130 °C, 3–24 h	29 62–91%	CHO, NO_2_, OMe	Br, Cl, NO_2_, OMe	
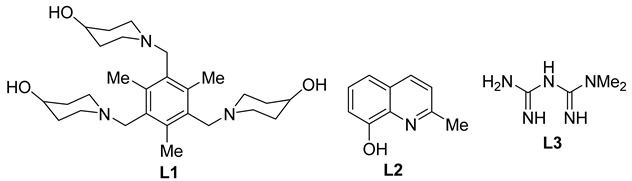					

**Table 3:** Copper-catalyzed *N*-arylation of indoles in the presence of a DMEDA ligand.

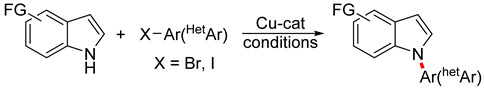					
Entry	Conditions	No. of Examples	^indole^FG		^Ar/hetAr^FG
1	CuO (2 equiv), K_2_CO_3_ (2 equiv), pyridine, reflux, 2–3 d	11 15–96%	Cl, F, CO_2_Et, OBn		CF_3_, F, OMe,
2	CuI (10 mol%), DMEDA (20 mol%), Cs_2_CO_3_ (1 equiv) DMSO, 120 °C, 4 h	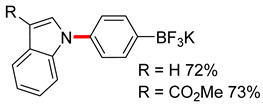			
3	CuI (10 mol%), DMEDA (20 mol%), K_3_PO_4_ (2 equiv), toluene, 110 °C, 24 h	13 74–91%		Ph	Cl, CO_2_Et, F, OMe
4	CuI (10 mol%), K_3_PO_4_ (3 equiv), DMEDA (20 mol%), toluene, 110 °C, 12 h	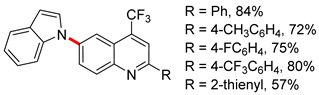			

**Table 4:** Copper-catalyzed *N*-arylations of indoles.

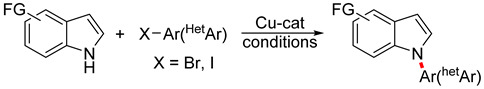				
Entry	Conditions	No. of Examples	^indole^FG	^Ar/hetAr^FG
1	CuI (10 mol%), DMSO (1 equiv), K_2_CO_3_ (2 equiv), glycerol, 120 °C, 24 h	12 65–92%	Br, NO_2_, OMe	Br, F, NO_2_, OMe
2	Cu (10 mol%), **L1** (20 mol%), Cs_2_CO_3_ (3 equiv) DMSO-H_2_O (1:1), 100 °C, 12–24 h	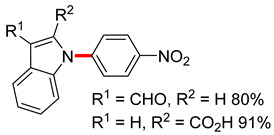		
3	Cu_2_O (10 mol%), **L2** (20 mol%), Cs_2_CO_3_ (2 equiv), DMSO, 120 °C, 3–24 h	15 86–97%	CH_3_, CHO	Cl, COMe, CO_2_Me, COCH_2_CO_2_Et OMe, NO_2_
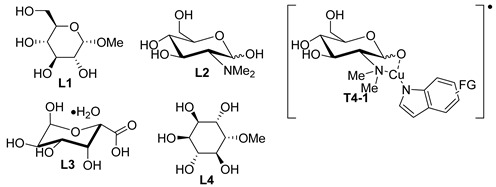				

**Table 5:** Copper-catalyzed *N*-arylation of indoles in water.

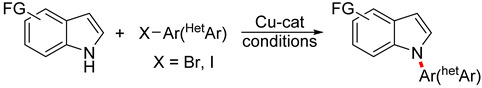				
Entry	Conditions	No. of Examples	^indole^FG	^Ar/hetAr^FG
1	CuBr_2_ (10 mol%), **L1** (10 mol%), D-glucose (10 mol%), *t*BuONa (2 equiv), TPGS-750-M (5 wt%), 50 °C, 24 h	11 43–95%	Br, Cl, F, OMOM, CHO, OMe, CH_2_CN, Cl, NO_2_ OMe	NO_2_, OMe
2	CuI (10 mol%), **L2** (20 mol%), K_3_PO_4_ (2 equiv), betaine (2 equiv.), water, 90 °C, 10 h	10 57–95%	–	Br, Cl, COMe, F, NO_2_, OMe
3	CuI (5 mol%), **L3** (10 mol%), KOH (2 equiv), DME-H_2_O (3:7), 95 °C, 20 h	23 42–95%	Br, CN, COMe, NO_2_	Br, Cl, I, OMe
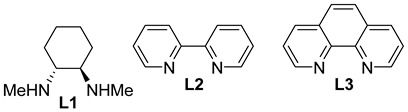				

**Table 6:** Pyridine- and phosphorous-based ligands for the *N*-arylation of indoles.

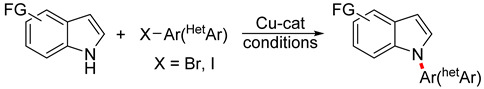				
Entry	Conditions	No. of Examples	^indole^FG	^Ar/hetAr^FG
1	Cu_2_O (10 mol%), **L1** (4 mol%), Cs_2_CO_3_ (2 equiv), DMSO, 120 °C, 20 h	6 72–95%	–	NO_2_, OMe
2	CuI (10 mol%), **L2** (10 mol%), K_2_CO_3_ (2 equiv), DMSO, 120 °C, 4–16 h	7 84–97%	–	NO_2_, OMe
3	CuI (10 mol%), **L3** (10 mol%), K_2_CO_3_ (1.4 equiv), DMSO, 110 °C, 24 h	4 69–99	CHO	OMe
4	CuCl (8 mol%), **L4** (16 mol%), NaOH (1 equiv), DMSO, 120 °C, 24 h	8 25–98%	–	Br, Cl, OEt, OMe
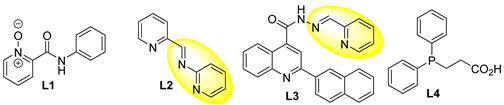				

## Data Availability

Not applicable.
